# Characterizing the Mechanisms of Metalaxyl, Bronopol and Copper Sulfate against *Saprolegnia parasitica* Using Modern Transcriptomics

**DOI:** 10.3390/genes13091524

**Published:** 2022-08-25

**Authors:** Yali Wang, Haotian Wu, Siying Fei, Junzhe Zhang, Kun Hu

**Affiliations:** 1 National Demonstration Center for Experimental Fisheries Science Education, Shanghai Ocean University, Shanghai 201306, China; 2 National Pathogen Collection Center for Aquatic Animals, Shanghai Ocean University, Shanghai 201306, China; 3 Key Laboratory of Freshwater Aquatic Genetic Resources, Ministry of Agriculture, Shanghai Ocean University, Shanghai 201306, China

**Keywords:** saprolegniasis, *Saprolegnia parasitica*, metalaxyl, bronopol, copper sulfate

## Abstract

Saprolegniasis, which is caused by *Saprolegnia parasitica*, leads to considerable economic losses. Recently, we showed that metalaxyl, bronopol and copper sulfate are good antimicrobial agents for aquaculture. In the current study, the efficacies of metalaxyl, bronopol and copper sulfate are evaluated by in vitro antimicrobial experiments, and the mechanism of action of these three antimicrobials on *S. parasitica* is explored using transcriptome technology. Finally, the potential target genes of antimicrobials on *S. parasitica* are identified by protein–protein interaction network analysis. Copper sulfate had the best inhibitory effect on *S. parasitica*, followed by bronopol. A total of 1771, 723 and 2118 DEGs upregulated and 1416, 319 and 2161 DEGs downregulated *S. parasitica* after three drug treatments (metalaxyl, bronopol and copper sulfate), separately. Additionally, KEGG pathway analysis also determined that there were 17, 19 and 13 significantly enriched metabolic pathways. PPI network analysis screened out three important proteins, and their corresponding genes were SPRG_08456, SPRG_03679 and SPRG_10775. Our results indicate that three antimicrobials inhibit *S. parasitica* growth by affecting multiple biological functions, including protein synthesis, oxidative stress, lipid metabolism and energy metabolism. Additionally, the screened key genes can be used as potential target genes of chemical antimicrobial drugs for *S. parasitica*.

## 1. Introduction

*Saprolegnia* is responsible for devastating infections in fish in aquaculture, fish farms and hobby fish tanks [1]. The infection of the *Saprolegnia* genus is characterized by white mycelial spots on the skin, gills and fins of the host. The mycelium produces and releases motile zoospores, which can germinate when attached to a new host after development and then form new mycelial mats [2,3]. *Saprolegnia parasitica* is, economically, one of the most important fish pathogens [1]. Outbreaks of this hard-to-control pathogen could cause tens of millions of dollars’ worth of economic losses to aquaculture businesses worldwide, notably in Scotland, Scandinavia, Chile, Japan, Canada and the USA [4]. In the past, *S. parasitica* infections were effectively controlled using malachite green. However, malachite green was banned worldwide in 2002 due to its carcinogenic and toxic effects [5]. To date, no valid substitutes have been discovered, thus there is an urgent need for novel alternative methods to manage saprolegniasis.

Metalaxyl is the world’s most widely used acetylalanine fungicide, which inhibits the synthesis of ribosomal RNA in mycelium [6]. Several studies have suggested that metalaxyl is an effective fungicide used for inhibiting *Phytophthora nicotianae*, *Phytophthora infestans* and *Phytophthora cinnamomi* in other plants [7,8,9]. Metalaxyl is also frequently used in aquaculture because of its significant inhibitory effect on fungi and its safety when targeting animals. The effective control of metalaxyl on fungal diseases suggests that it may be an effective drug for the prevention and treatment of *S. parasitica*.

Bronopol (2-bromo-2-nitro-1,3-propandiol) is an alcohol produced by the bromination of di(hydroxymethyl)nitromethane [10]. In the manufacturing industry, bronopol is commonly used in agricultural fungicides, cosmetic preservatives, water-treatment fungicides and petroleum industry fungicides [11,12,13]. It has been discovered that the number of bacteria on tilapia eggs could be significantly reduced when the fertilized tilapia eggs were soaked in bronopol for a certain period of time [14]. In a study conducted on rainbow trout, the incidence of fungal infection in the eggs of rainbow trout decreased after being treated with 100 mg/L of bronopol for 30 min [15]. These results suggest that bronopol may be a substitute for malachite green.

Copper sulfate is a salt with high solubility in water. It can be used as an antifungal fungicide, as well as for the prevention and control of animal foot rot disease and the removal of pond algae [16]. The combination of copper sulfate and FeSO_4_·7H_2_O and refined trichlorfon powder had a remarkable killing effect on two parasites (*Dactylogyrus* and *Chilodonella cyprini*). In addition, copper sulfate is effective in controlling the mycelium growth of largemouth bass eggs (caused by *Sapproplegnia* spp.) and improving fry survival in mat spawning [17].

It is evident from the information above that metalaxyl, bronopol and copper sulfate can be used as alternatives to malachite green for saprolegniasis caused by *S. parasitica*. Metalaxyl and copper sulfate have been shown in some reports to be effective in inhibiting the growth of *S. parasitica* [18,19]. However, the mechanism of inhibition of *S. parasitica* growth by metalaxyl and copper sulfate is unclear and it is necessary to study it further. No reports have been discovered that highlight that bronopol can inhibit the growth of *S. parasitica*, and further studies are required to prove this. In the current study, the efficacies of metalaxyl, bronopol and copper sulfate are evaluated by an in vitro inhibiting test, and the mechanism of action of these three antimicrobials on *S. parasitica* is explored using transcriptome technology. This provides a theoretical basis for the subsequent screening of effective drugs against *S. parasitica*.

## 2. Materials and Methods

### 2.1. Determination of MIC and MBC of Three Antimicrobial Agents

*S. parasitica* (ATCC 200013) was obtained from American Type Culture Collection (Manassas, VA, USA) and cultured in potato dextrose agar (Sinopharm Chemical Reagent Co., Ltd., Shanghai, China).

The metalaxyl, bronopol and copper sulfate used in this study were purchased from Guoyao Chemical Reagent (Shanghai, China). To determine the minimum inhibitory concentration (MIC) and minimum bactericidal concentration (MBC) of the three antimicrobials, different concentrations of the concerned antimicrobials (0.0, 1.0, 2.0, 3.0, 4.0, 5.0, 6.0, 7.0, 8.0, 9.0, 10.0, 15.0, 20.0, 25.0 and 30.0 mg/L) were prepared by dissolving their requisite amount in water. MIC and MBC were also determined by using the microdilution method and the results were also expressed in mg/L.

### 2.2. Total RNA Isolation

*S. parasitica* cultures were prepared in potato dextrose broth to an initial optical density at 600 nm (OD_600_) of 0.05 and then incubated in a 250 mL cell culture flask (Falcon; Corning, Corning, NY, USA) at 20 °C for 4 days with shaking at 120 rpm. The cultures were then split into three flask containing 2 mL of each treatment at a final concentration of three antimicrobials: metalaxyl (5.0 mg/L), bronopol (4.0 mg/L) and copper sulfate (2 mg/L). A fifth flask containing 2 mL of fresh liquid potato dextrose agar remained untreated. Cultures were grown for an additional 30 min before lysis reagent (Sangon Biotech, Shanghai, China). Total RNA was isolated using TRIzol reagent (Invitrogen, Waltham, MA, USA) according to the manufacturer’s instructions. Experiments were conducted in triplicate and samples were removed from DNA contaminants following treatment with RNasefree DNase I (Takara Biotechnology, Dalian, China). RNA integrity was confirmed using an Agilent 2100 Bioanalyzer (Agilent, Santa Clara, CA, USA) with all samples having RNA integrity > 9.0.

### 2.3. RNA Sequencing (RNA-seq) Library Construction and Sequencing

Poly-(A) mRNA was enriched from total RNA using Oligo-(dT) magnetic beads, then fragmented using a fragmentation buffer. Cleaved RNA fragments were copied into first-strand complementary DNA (cDNA) with reverse transcriptase and random hexamer primers, followed by second-strand cDNA synthesis using DNA polymerase I and RNase H. The cDNA fragments underwent an end-repair process and poly(A) was added and then ligated with the Illumina paired-end sequencing adaptors. The products were purified and enriched with a polymerase chain reaction (PCR) to create the final cDNA sequencing library. Following validation on an Agilent 2100 Bioanalyzer and ABI StepOnePlus RealTime PCR System (ABI, Los Angeles, CA, USA), the cDNA library was sequenced on a flow cell using high-throughput 101-bp paired-end mode on an Illumina HiSeq 2500 (Illumina, San Diego, CA, USA) unit.

### 2.4. Sequencing, Data Processing and Quality Control

The RNA-Seq read quality was assessed using FASTQC (version 0.11.5) and trimmed using Trimmomatic (version 0.36) with default parameters and trimmed of adaptor sequences (TruSeq3 paired-ended). Subsequently, we conducted a comparative analysis with the reference genome (ASM15154v2). For each sample, sequence alignment with the reference genome sequences was performed using Tophat [20].

### 2.5. The Analysis of Differential Expression Genes (DEGs)

HTSeq (version 0.6.1) (https://pypi.python.org/pypi/HTSeq (accessed on 11 April 2022)) was used to count the read numbers mapped to each gene [21]. The FPKM (fragments per kilobase of transcript per million mapped reads) method was used for estimating gene expression levels [22]. Generally, when FPKM > 0.1, this indicated that a given transcript was expressed. Meanwhile, the Cuffdiff program was used to calculate RNAs that were differentially expressed in reprogramming, using a fold change > 2 and *p* < 0.05.

### 2.6. DEG-Enrichment Analysis

GO-enrichment analysis (with Goatools, https://github.com/tanghaibao/Goatools (accessed on 15 April 2022)) of the differentially expressed genes was performed, and their function was described (in combination with the GO annotation results). KEGG analysis was performed using the KEGG pathway database (http://kobas.cbi.pku.edu.cn/home.do (accessed on 15 April 2022)).

### 2.7. Protein Network Analysis of DEGs

Protein–protein interaction (PPI) was constructed based on the corresponding protein information of genes to obtain information on the known and anticipated gene interactions [23,24]. STRING database (http://string-db.org/ (accessed on 1 May 2022)) was used to analyze the protein–protein interaction network of the genes of interest. After the protein–protein interaction network relationship was obtained, the NetworkX library in Python was used to visualize the network of interested genes.

### 2.8. RT qPCR

To validate the RNA-seq data, 10 genes (see details in Supplementary Table S1) were selected for confirmation by quantitative RT-PCR (qRT-PCR) assays. Primers were designed using Primer 5.0 software (Premier company, Burnaby, BC, Canada), and SpTub-b (*S.parasitica* Tub-b) was used as the reference gene [25,26]. The thermal cycling program was 95 °C for 30 s, followed by 40 cycles of 95 °C for 5 s, 60 °C for 30 s and 72 °C for 30 s.

The reactions were performed in a 25 μL volume, composed of 2 μL cDNA, 0.5 μL of both the forward and reverse primers (10 μM), 12.5 μL SYBR Premix Ex Taq (2×) and 9.5 μL RNase-free H_2_O. The reaction procedure was performed as follows: 95 °C for 30 s, followed by 40 cycles of 95 °C for 5 s, 60 °C for 30 s and 72 °C for 30 s. The 2^−ΔΔCT^ method was used for calculations, and each sample was examined in triplicate [27].

## 3. Results

### 3.1. The Antimicrobial Activity of Three Antimicrobials against S. parasitica

We determined the contribution of three antimicrobials to the antimicrobial activity against *S. parasitica* by minimum inhibitory and minimum bactericidal concentration (MIC and MBC, respectively) assays (Table 1). We observed that copper sulfate had the best inhibitory effect on saprolegniasis, followed by bronopol.

### 3.2. Illumina Sequencing and Quality Assessment

A total of 12 cDNA libraries were constructed and sequenced. The RNA sequencing generated a total of 639,148,432 raw reads (162,407,898 in the control group, 168,335,074 in the metalaxyl group, 150,030,784 in the bronopol group and 158,374,676 in the copper sulfate group) and 278,935,201 clean reads (161,022,918 in the control group, 166,799,912 in the metalaxyl group, 148,629,810 in the bronopol group and 633,536,914 in the copper sulfate group) were obtained after removing TruSeq adaptors and low-quality reads. The Phred-like quality scores of the clean reads at the Q30 level ranged from 93.71–96.96% and the average GC content was estimated as 61.71% (see details in Supplementary Table S2). Among those reads, 84.72–88.4% of the clean reads were mapped to the reference genome, and 79.11–81.84% were only mapped to unique positions in the reference genome (see details in Supplementary Table S3).

### 3.3. Effects of Three Antimicrobials on the Genome Expression of S. parasitica

**Figure 1:**
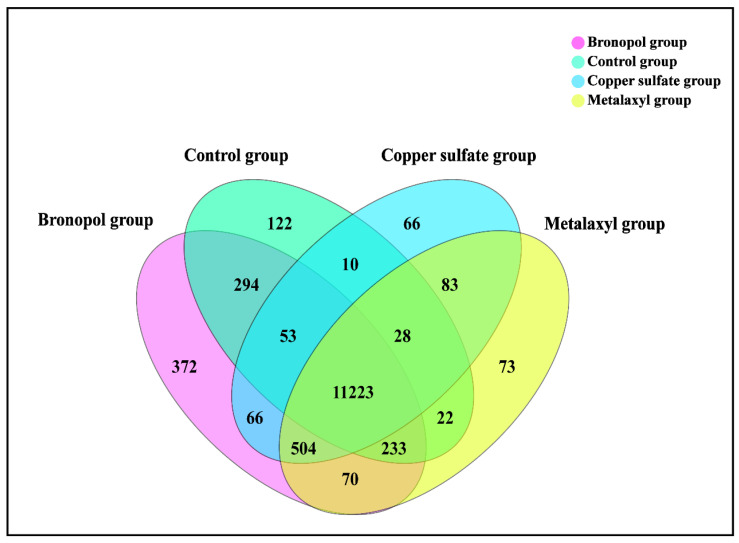
Venn diagram of expressed genes in *Saprolegnia parasitica* following treatment with metalaxyl, bronopol and copper sulfate at MICs.

To investigate the gene contents and expression patterns associated with the *S. parasitica* following treatment with metalaxyl, bronopol and copper sulfate, we compared and characterized the control, metalaxyl, bronopol and copper sulfate groups’ expressed genes. The expression of genes was analyzed in each group. A total of 11,985, 12,236, 12,815 and 12,033 genes had expression levels greater than 1 TPM in the control, metalaxyl, bronopol and copper sulfate groups, respectively. The expression of roughly 11,223 genes was shared by the four groups in *S. parasitica*. On the other hand, there were 122, 372, 73 and 66 specific genes expressed in the control, metalaxyl, bronopol and copper sulfate groups, respectively (Figure 1).

**Figure 2:**
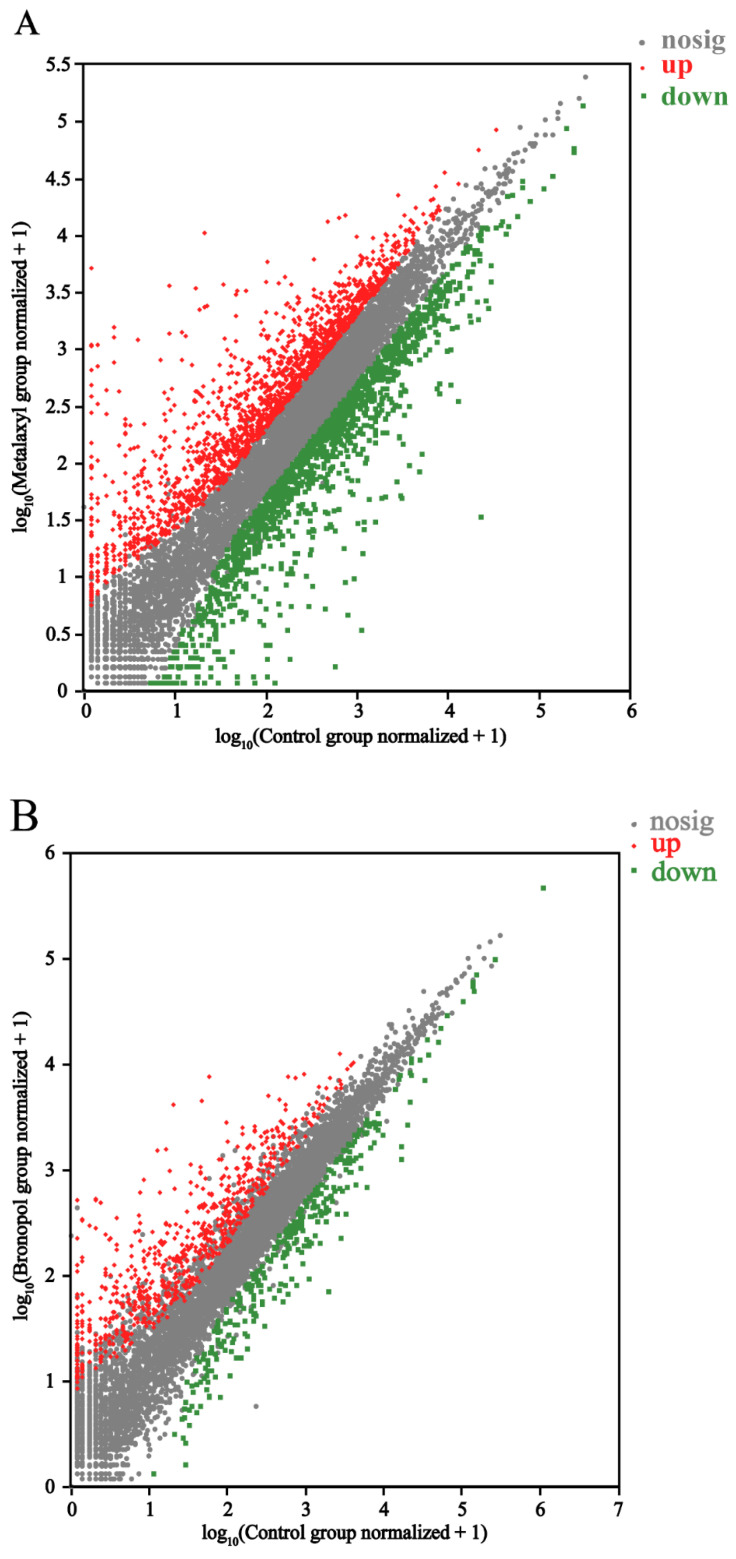

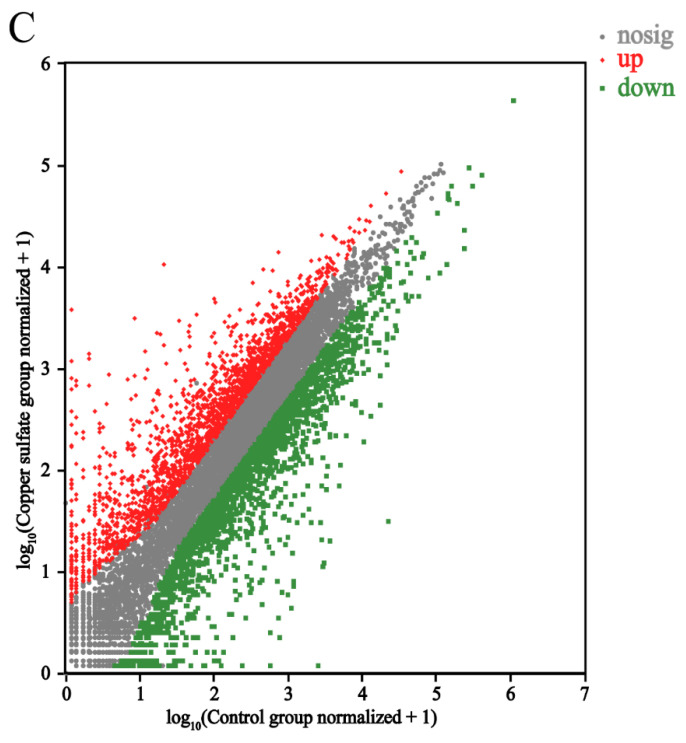
Scatter plots of gene expression in *Saprolegnia parasitica* following treatment with metalaxyl (**A**), bronopol (**B**) and copper sulfate (**C**). Gray dots indicate genes with no significant difference compared to the untreated control (*P_adj_* < 0.05), green dots indicate significantly downregulated genes compared to the untreated control (*P_adj_* < 0.05) and red dots indicate significantly upregulated genes (*P_adj_* < 0.05) and log2FC of >2 compared to the untreated control, with numerical annotations to indicate the number of differentially expressed genes.

**Figure 3:**
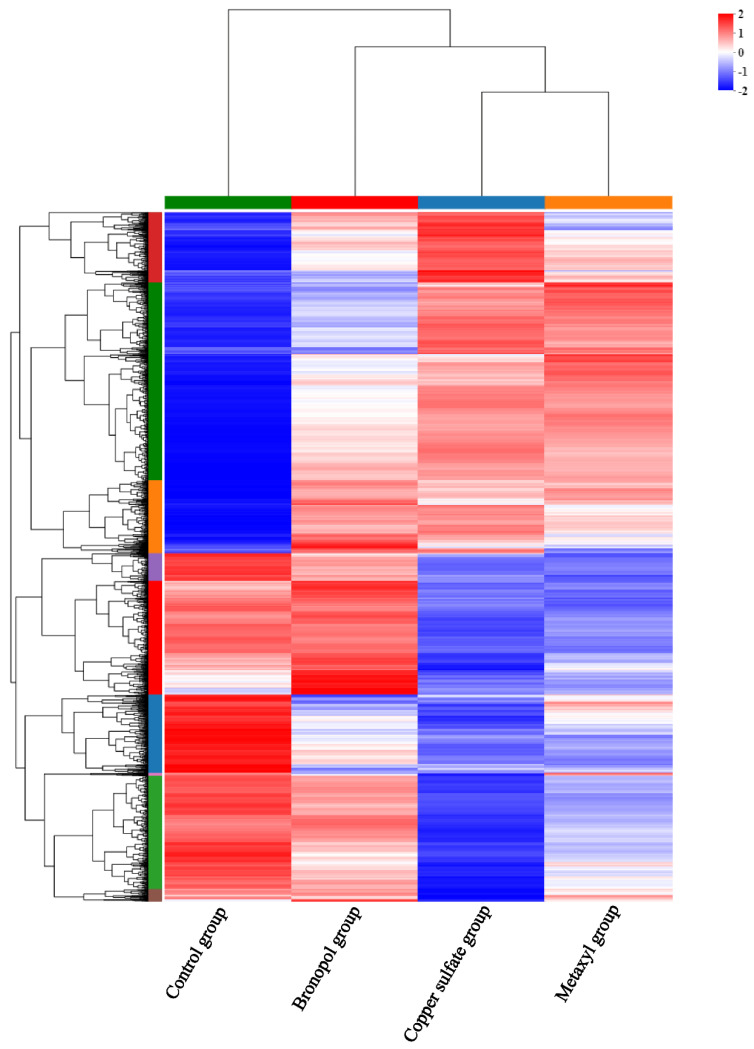
Heatmap presents the gene expression profile for *Saprolegnia parasitica* treated with metalaxyl, bronopol and copper sulfate. Red: upregulated genes; green: downregulated genes. Each line represents one gene.

Genes of *S. parasitica* following three antimicrobials’ action were screened (analyses of the screening data). Three groups of data were compared: control vs. metalaxyl, control vs. bronopol and control vs. copper sulfate groups. In total, 8508 DEGs were identified upon pairwise comparisons: 3187 DEGs (1771 up- and 1416 downregulated) in the control vs. metalaxyl group, 1042 DEGs (723 up- and 319 downregulated) in the control vs. bronopol group and 4279 DEGs (2118 up- and 2161 downregulated) in the control vs. copper sulfate group (Figure 2). Unsupervised cluster analyses were performed based on DEGs using a Euclidean distance formula. In comparison to the control group, the gene expressions of *S. parasitica* were significantly affected by the antimicrobials. There were also significant differences among the metalaxyl, bronopol and copper sulfate groups, which indicated that the three antimicrobials had different effects on *S. parasitica* (Figure 3).

### 3.4. Functional Annotation of DEGs

**Figure 4:**
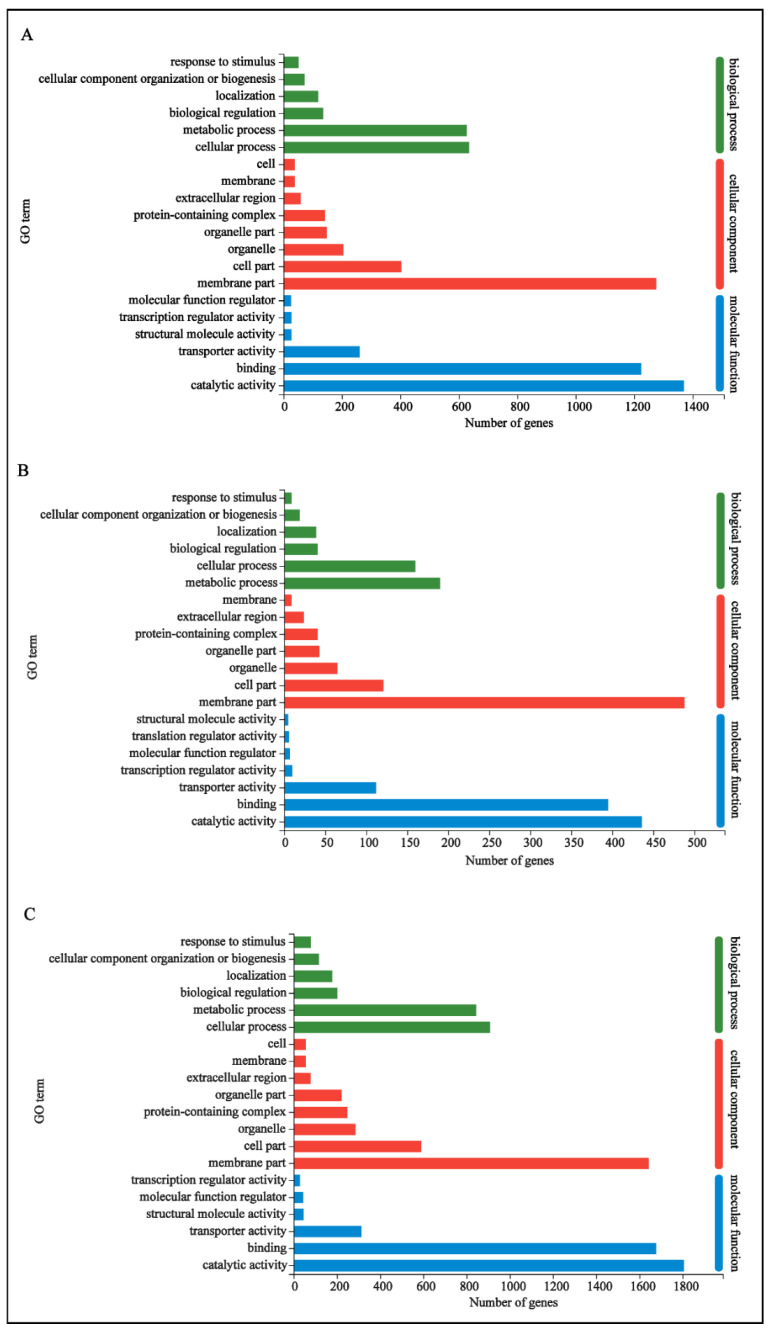
DEGs of control vs. metalaxyl group (**A**), control vs. bronopol group (**B**) and control vs. copper sulfate group (**C**) were assigned to GO categories, and the terms are categorized into three main GO categories. The top 20 enriched GO terms are presented in the figure.

The DEGs were characterized using the GO and KEGG databases. In the control vs. metalaxyl group, 15 terms for biological processes, 10 terms for cellular components and 11 terms for molecular functions were annotated (see details in Supplementary Table S4). The DEGs were significantly enriched in the cellular process, metabolic process, membrane part, catalytic activity and binding (Figure 4A). In the control vs. bronopol group, nine terms for biological processes, nine terms for cellular components and seven terms for molecular functions were annotated (see details in Supplementary Table S5). The DEGs were significantly enriched in the metabolic process, cellular process, membrane part, catalytic activity, binding and transporter activity (Figure 4B). In the control vs. copper sulfate group, 15 terms for biological processes, 10 terms for cellular components and 13 terms for molecular functions were annotated (see details in Supplementary Table S6). The DEGs were significantly enriched in cellular process, metabolic process, membrane part, catalytic activity and binding (Figure 4C). According to GO annotation analysis, the DEGs of the three groups were significantly enriched in the cellular process, metabolic process, membrane part, catalytic activity and binding, which showed that the three antimicrobial agents could inhibit the growth of *S. parasitica* mainly through five GO terms, and different antimicrobial had different effects on different processes. Furthermore, to analyze the involved signal pathways, the DEGs were annotated in the KEGG database. A number of 548 DEGs in the control vs. metalaxyl group were assigned to 100 KEGG pathways. Among them, amino acid metabolism (including 14 pathways), carbohydrate metabolism (including 13 pathways) and lipid metabolism (including 13 pathways) contained more DEGs. A number of 167 DEGs in the control vs. bronopol group were assigned to 75 KEGG pathways. Lipid metabolism (including 13 pathways), amino acid metabolism (including 12 pathways) and carbohydrate metabolism (including 12 pathways) contained more DEGs. Finally, a certain number of the 673 DEGs in the control vs. copper sulfate group were assigned to 106 KEGG pathways. The DEGs were significantly enriched in amino acid metabolism (including 14 pathways), carbohydrate metabolism (including 14 pathways) and lipid metabolism (including 14 pathways). These annotations are useful to identify functional genes and specific biological processes in *S. parasitica.* Further information is presented in Supplementary Tables S7–S9.

### 3.5. Enrichment Analysis of DEGs

**Figure 5:**
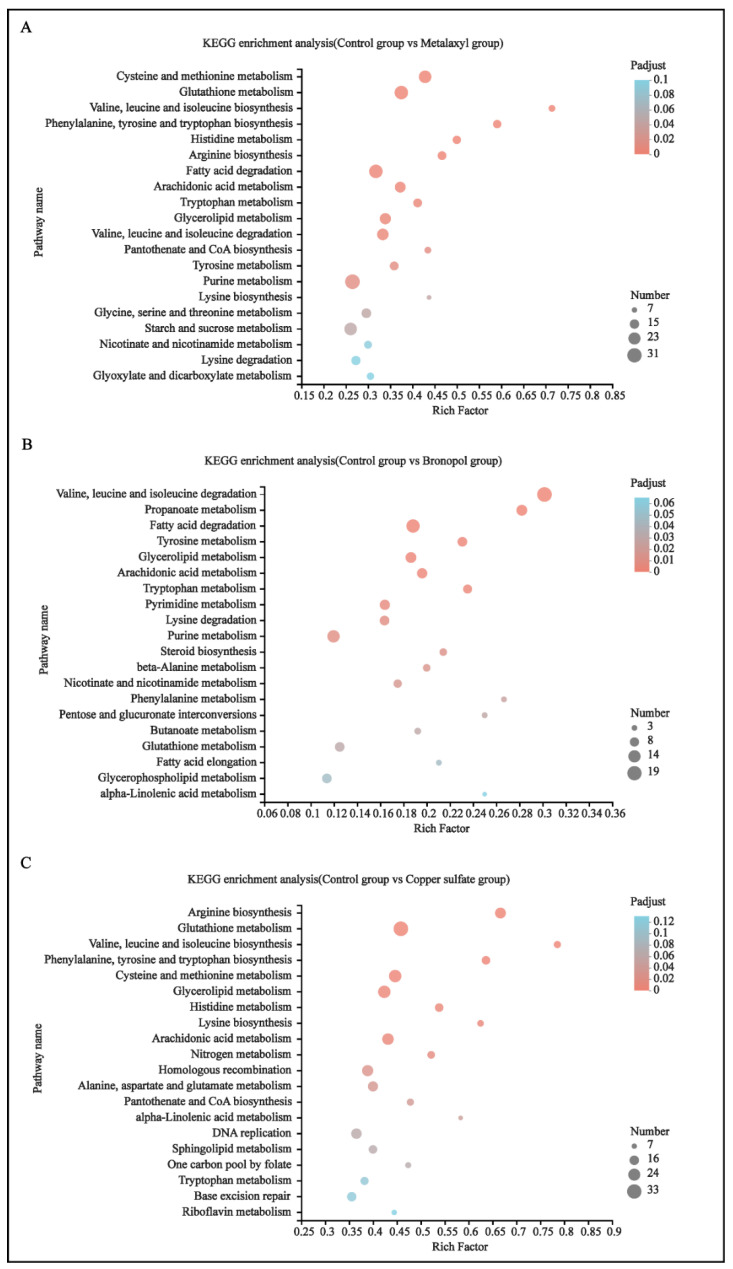
DEGs of control vs. metalaxyl (**A**), control vs. bronopol (**B**) and control vs. copper sulfate groups (**C**) were assigned to KEGG pathway annotations. The top 20 enriched KEGG pathways are presented in the figure.

KEGG-enrichment analysis was performed with a *p* < 0.05. For the control vs. metalaxyl group, 17 pathways were identified as significantly enriched pathways, focusing on metabolic pathways. These pathways included 11 amino acid metabolic pathways, 3 lipid metabolic pathways, 1 cofactor and vitamin metabolic pathway, 1 nucleotide metabolic pathway and 1 carbohydrate metabolic pathway, among which cysteine and methionine metabolism (map00270); glutathione metabolism (map00480); valine, leucine and isoleucine biosynthesis (map00290); phenylalanine, tyrosine and tryptophan biosynthesis (map00400) and histidine metabolism (map00340) pathways presented the most significant differences (Figure 5A and Table S10). From the five significant differences, there were 24 DEGs enriched in the cysteine and methionine metabolism pathway, mainly including the PLP-dependent enzyme, pyridoxal-phosphate dependent enzyme and aminotransferase classes I and II; 27 DEGs enriched in the glutathione metabolism pathway, mainly including glutathione S-transferase, the MAPEG family and ribonucleotide reductase; 10 DEGs enriched in the valine, leucine and isoleucine biosynthesis pathway, mainly including the dehydratase family, amino-transferase class IV, pyridoxal-phosphate dependent enzyme and acetohydroxy acid isomeroreductase; 13 DEGs enriched in phenylalanine, tyrosine and tryptophan biosynthesis pathways, mainly including chorismate synthase, DAHP synthetase I family and glycosyl transferase family; and 13 DEGs enriched in the histidine metabolism pathway, mainly including ATP phosphoribosyltransferase, the aldehyde dehydrogenase family and histidine biosynthesis protein. Further information is presented in Supplementary Figures S1–S5 and Tables S11–S15.

For the control vs. bronopol group, 19 pathways were identified as significantly enriched pathways. These 19 pathways also focused on the metabolic pathway, including 7 amino acid metabolic pathways; 3 carbohydrate metabolic pathways; 6 lipid metabolic pathways; 2 nucleotide metabolic pathways and 1 cofactor and vitamins metabolic pathway, among which valine, leucine and isoleucine degradation (map00280); propanoate metabolism (map00640) and fatty acid degradation (map00071) pathways presented the most significant differences (Figure 5B and Table S10). In three significant differences, there were 19 DEGs enriched in the valine, leucine and isoleucine degradation pathways, mainly including the carbamoyl-phosphate synthase L chain, ATP binding domain and thiolase; 11 DEGs enriched in the propanoate metabolism pathway, mainly including the carbamoyl-phosphate synthase L chain, ATP binding domain and 2-oxoacid dehydrogenases acyltransferase; and 16 DEGs enriched in the fatty acid degradation pathway, mainly including the AMP-binding enzyme, thiolase and N-terminal domain. Further information is presented in Supplementary Figures S6–S8 and Tables S16–S18.

For the control vs. copper sulfate group, 14 pathways were identified as significantly enriched pathways. Among these 14 pathways, 13 pathways were metabolic pathway and 1 pathway was genetic information processing. These 13 metabolic pathways were, respectively, eight amino acid metabolic pathways; three lipid metabolic pathways; one energy metabolic pathway and one cofactors and vitamins metabolic pathway, among which arginine biosynthesis (map00220) and glutathione metabolism (map00480) pathways presented the most significant differences (Figure 5C and Table S10). In four significant differences, 20 DEGs were enriched in the arginine biosynthesis pathway, mainly including the amino acid kinase family and peptidase family M20/M25/M40, aminotransferase class III; 33 DEGs were enriched in the glutathione metabolism pathway, mainly including glucose-6-phosphate dehydrogenase, C-terminal domain and glutathione S-transferase; 11 DEGs were enriched in valine, leucine and isoleucine biosynthesis pathways, mainly including the dehydratase family, amino-transferase class IV and pyridoxal-phosphate-dependent enzyme; 13 DEGs were enriched in phenylalanine, tyrosine and tryptophan biosynthesis pathways, mainly including chorismate synthase, DAHP synthetase I family and glycosyl transferase family; and 13 DEGs were enriched in the histidine metabolic pathway, mainly including ATP phosphoribosyltransferase, aldehyde dehydrogenase family and histidinol dehydrogenase. Further information is presented in Supplementary Figures S9–S12 and Tables S19–S22.

The results above show that the pathways significantly enriched in control vs. metalaxyl, control vs. bronopol, and control vs. copper sulfate groups were all metabolic pathways, among which the amino acid metabolic pathway was the most significant and contained the most pathways, suggesting that the three antimicrobial agents could inhibit *S. parasitica* mainly by disrupting the amino acid metabolism. The results provide a direction for further analysis of the mechanism of action of the three drugs.

### 3.6. Protein Network Analysis

**Figure 6:**
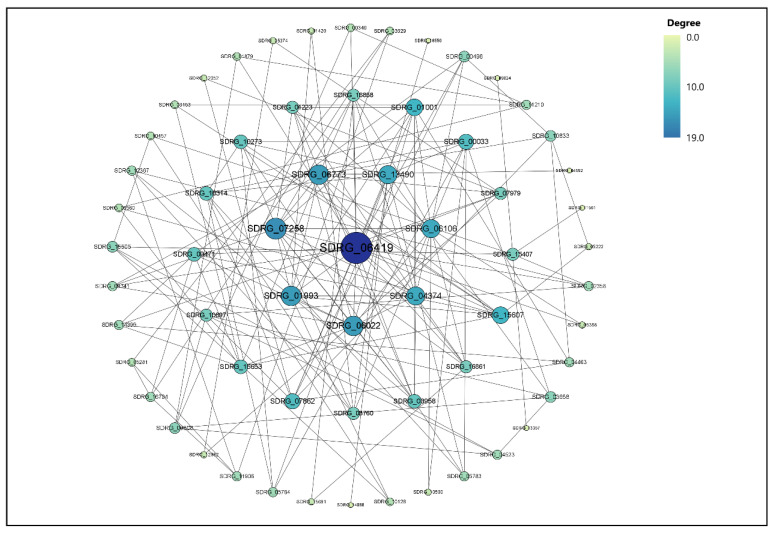
The DEGs in the protein–protein interaction networks are presented as nodes. Node’s name is the protein’s name. The size and color of the node are proportional to the degree of the node.

Based on the results of transcriptome sequencing, 314 DEGs from control vs. metalaxyl, control vs. bronopol and control vs. copper sulfate groups were screened for protein–protein interaction network analysis. The 314 DEGs were present in control vs. metalaxyl, control vs. bronopol and control vs. copper sulfate groups and were significantly different (*p* < 0.05). The main protein interaction cluster derived from the 314 DEGs contained 63 nodes, each representing 1 protein and connected by 100 edges (Figure 6). SDRG_06419 (SPRG_08456), followed by SDRG_07258 (SPRG_03679), SDRG_01993 (SPRG_04185), SDRG_06022 (SPRG_10775) and SDRG_06773 (SPRG_05141) presented the highest scores for degree centrality (DC), indicating that they were the most important factors for the network.

### 3.7. Verification of DEGs by qRT-PCR

**Figure 7:**
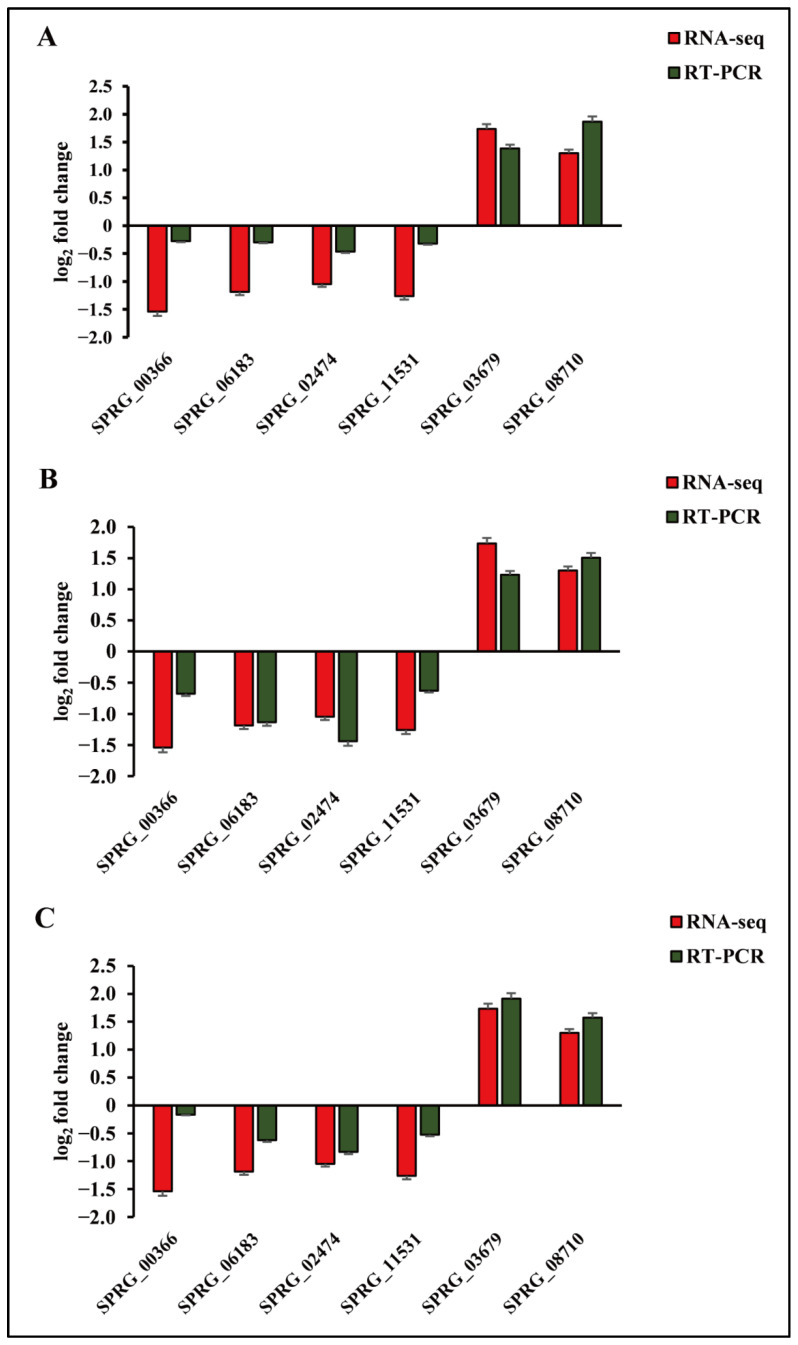
Comparison of RNA-seq results and qRT-PCR analysis of gene expression levels. (**A**) Log_2_ fold change of 6 genes for control vs. metalaxyl (**A**), control vs. bronopol (**B**), and control vs. copper sulfate groups (**C**).

To validate the expression data of RNA-seq, we selected six DEGs for qRT-PCR analysis, including two upregulated (SPRG_03679, SPRG_08710) and four downregulated (SPRG_00366, SPRG_06183, SPRG_02474, SPRG_11531) genes (Figure 7; Supplementary Table S1). To compare the expression data between RNA-seq and qRT-PCR, the relative expression level was transformed to log_2_ fold change. The results verify that six of the genes examined are consistent with the results of RNA-Seq, indicating the reliability of the RNA-seq expression profile in this study.

## 4. Discussion

Saprolegniasis causes a considerable economic loss to aquaculture. However, there are few effective drugs to control saprolegniasis. Metalaxyl, bronopol and copper sulfate are widely used in agriculture as antiparasitic, antibacterial and antifungal agents. Therefore, we studied the effect of these three drugs against *S. parasitica*, and the results show that the three antimicrobial agents can effectively inhibit the growth of *S. parasitica*. Subsequently, transcriptomics were used to study the core genes and metabolic pathways of the drug-acting *S. parasitica*, providing important information for an in-depth study of the molecular mechanism of drug-treated *S. parasitica*. At the same time, three key genes were screened out, which could be used as potential target genes of chemical antimicrobial drugs against *S. parasitica*.

Transcriptome experiments showed that, compared to the other two drugs, after copper sulfate treatment, the genes and metabolic pathways were changed most in *S. parasitica*. At the same time, in vitro antimicrobial experiments also confirmed that copper sulfate has the strongest inhibition effect. These results suggested that copper sulfate had good control of *S. parasitica* and could be used as a drug additive to prevent *S. parasitica* infections in aquaculture.

Through the GO database annotation, it was observed that the DEGs of three groups (control vs. metalaxyl, control vs. bronopol and control vs. copper sulfate) in the biological processes were mainly distributed in the cellular and metabolic processes. Additionally, the KEGG database annotation determined that DEGs in the three groups were mainly distributed in the metabolic pathways, similar to the results of biological process enrichment in the GO database. According to the above results, we mainly discuss the effects of the three drugs on the metabolic pathway of *S. parasitica*.

Following the treatment of *S. parasitica* with metalaxyl, cysteine and methionine metabolism (map00270); glutathione metabolism (map00480); valine, leucine and isoleucine biosynthesis (map00290); phenylalanine, tyrosine and tryptophan biosynthesis (map00400) and histidine metabolism (map00340) pathways were observed to present the most significant differences (Figures S1–S5 and Tables S11–S15). Map00270, map00480 and map00340 can affect the oxidative stress response of *S. parasitica* through protein metabolism and the normal function of the immune system. Methionine is a conversion of L-cysteine synthesized during sulfur metabolism and is extremely sensitive to reactive oxygen species (ROS), producing methionine sulfoxide (MetO) to improve oxidative damage [28,29,30,31]. In addition, methionine can effectively regulate the metabolic process and immune system, activate endogenous antioxidant enzyme activity and synthesize glutathione [32]. Glutathione metabolism fights oxidative stress by increasing glutathione S-transferase (GST), peroxidase (PRDX) and superoxide dismutase (SOD) [33]. Histidine, as a glucose amino acid, can maintain the balance of oxidative metabolism through gluconeogenesis and glycolysis [34]. In our study, methionine, glutathione and histidine contents in *S. parasitica* were downregulated by metalaxyl action, which can cause oxidative damage, and thus destroy cellular molecular functions. The disturbance of these pathways is mainly caused by the significant regulation of the genes enriched in them. Pyridoxal 5′-phosphate (PLP) is a versatile catalyst, acting as a coenzyme in a multitude of reactions. Pyridoxal-dependent enzymes are involved in cysteine, homocysteine, methionine and other metabolic processes. Aminotransferase functions similar to pyridoxal-phosphate-dependent enzymes and is involved in pyridoxal-phosphate-dependent enzymes and biological binding processes. On the basis of sequence similarity, these various enzymes can be grouped into classes I and II [35]. It has been reported that aspartate and homoserine are the precursors of methionine synthesis, and their biosynthesis is mainly controlled by hemialdehyde dehydrogenase and α/β hydrolase families, respectively [36,37]. Similarly, the S-adenosine-L-homocysteine hydrolases and family of amino acid kinases can play a key role in regulating intracellular concentrations of adenosine homocysteine and methionine [38]. The downregulation of these related genes indicated that the metabolism of amino acids, such as cysteine and methionine, was blocked, resulting in oxidative damage. Leucine, valine and isoleucine in map00290, collectively known as branched chain amino acids (BCAAs), can be significant to protein synthesis, immune function and oxidative energy supply [39,40,41]. Leucine, as a regulator of intracellular signaling pathways, can significantly increase protein synthesis and promote the release of related hormones (such as growth hormone (GH)) [42]. Phenylalanine, tryptophan and tyrosine in map00400 belong to the aromatic amino acid family and are important sources of benzene metabolites. These metabolites play a key role in the biosynthesis of other amino acids [43]. The degradation of valine, leucine and isoleucine was generally upregulated and biosynthesis was generally downregulated in the enrichment pathways, indicating that the protein decomposition process was accelerated, but the synthesis process was inhibited, leading to a decrease in protein levels. The overall downregulation of phenylalanine, tyrosine and tryptophan biosynthesis can lead to disturbances in other metabolic pathways. The disruption of the pathway is mainly due to the significant changes in the genes enriched in it. In eukaryotes, GSTs participate in the detoxification of reactive electrophilic compounds by catalyzing their conjugation to glutathione [44]. This suggests that relevant genes can be involved in the detoxification process. In molecular biology, the MAPEG (membrane-associated proteins in eicosanoid and glutathione metabolism) family of proteins are a group of membrane-associated proteins with highly divergent functions [45]. The downregulation of related genes can affect glutathione synthesis and damage cell membranes, which is consistent with the previous GO analysis. Ribonucleotide reductase (RNR) plays a critical role in regulating the total rate of DNA synthesis so that the ratio of DNA to cell mass remains constant during cell division and DNA repair [46]. The downregulation of genes encoding RNR can trigger deoxyribonucleotide pool imbalance and DNA damage. Acetohydroxy acid isomeroreductase catalyzes the conversion of acetohydroxy acids into dihydroxy valerates. This reaction is the second in the synthetic pathway of the essential branched side chain amino acids valine and isoleucine [47]. The downregulation of these related genes can prevent reduced BCAA levels. Based on the above findings, we observed that metalaxyl can affect the synthesis and degradation of various amino acids, enzyme content and cell structure, thereby inhibiting protein synthesis, metabolism, neurotransmitter transport and biosynthesis, and DNA synthesis.

Following *S. parasitica* treatment with bronopol; valine, leucine and isoleucine degradation (map00280); propanoate metabolism (map00640) and fatty acid degradation (map00071), the pathways presented the most significant differences (Figures S6–S8 and Tables S16–S18). Map00280 was mainly involved in protein synthesis and the energy metabolism of *S. parasitica*. The functions of valine, leucine and isoleucine were described in the previous paragraph; therefore, the description is not repeated in detail here. The genes enriched in this pathway were significantly different. Methylmalonyl-CoA mutase (MCM) is an adenosylcobalamin-dependent enzyme that catalyzes the interconversion of (2R)-methylmalonyl-CoA to succinyl-CoA [48]. Methylmalonyl-CoA is an anaplerotic substrate mainly derived from the metabolism of branched-chain amino acids (i.e., valine and isoleucine) and enters into the citrate cycle (TCA cycle)) [49]. The upregulation of MCM-expressed genes leads to the accumulation of methylmalonyl-CoA and toxic metabolites of other compounds, blocking circulation [50]. Throughout our study, we observed that the related genes were upregulated. Bronopol treatment of *S. parasitica* led to the reduced uptake or mobilization of amino acids from intracellular protein stores, resulting in reduced TCA cycle production and energy [51]. The fatty acid metabolism and oxidative stress reaction of *S. parasitica* are affected by map00640 and map00071. Fatty acid degradation is an important metabolic pathway in organisms, and the growth of microorganisms requires fatty acids, but at the same time, some bacteria are toxic to other bacteria due to their varying sensitivity to fatty acid, so it has been proposed that fatty acid sterilization is a protective mechanism [52]. The widespread upregulation of both pathways is related to the inhibitory effect of bronopol on *S. parasitica*. Simultaneously, the enriched genes in the pathway also confirmed this view. Acyl-CoA dehydrogenase (ACAD) can catalyze the β-oxidation of fatty acids and is the main source of ROS [53]. The upregulation of the ACAD gene leads to ROS production, and the imbalance between ROS production and the antioxidant system can promote DNA damage, lipid peroxidation and cell death [53,54]. Enoyl-CoA hydratase, an important participant in the β-oxidation of fatty acids, is a key enzyme of *S. parasitica*. Enoyl-CoA hydratase is not only involved in the β-oxidation of fatty acids, but is also associated with lysine degradation, isoleucine degradation, valine degradation, tryptophan metabolism and β-alanine metabolism. As an enzyme related to fatty acid synthesis and degradation, it is an ideal target for screening antimicrobial drugs [55]. 3-hydroxyl-CoA dehydrogenase (HADHA) is a key enzyme in fatty acid β-oxidation, which is involved in the removal of two carbon atoms from long-chain fatty acids. Moreover, HADHA can be used as an effective candidate drug for the development of new vaccines because it can induce partial immune protection against pathogen infection [56]. Therefore, bronopol considerably affects the oxidative decomposition of fatty acids, changes the degradation of BCAAs and inhibits protein synthesis and energy supply. Bronopol presents good potential as a treatment against *S. parasitica*.

Following *S. parasitica* treatment with copper sulfate, arginine biosynthesis (map00220); glutathione metabolism (map00480); valine, leucine and isoleucine biosynthesis (map00290) and phenylalanine, tyrosine and tryptophan biosynthesis (map00400) present the most significant differences (Figures S9–S12 and Tables S19–S22). The four pathways had a considerable influence on the energy metabolism of *S. parasitica*. Arginine biosynthesis (map00220) can be observed in bacteria, plants and fungi [57]. Arginine is an intermediate of the urea cycle, exerting a crucial role in regulating energy metabolism processes and amino acids with the highest number of nitrogen atoms [58]. Map00480, map00290 and map00400 have been described in the first two groups, so we only briefly describe them here. In our study, conditions for arginine biosynthesis and glutathione metabolism are no longer favorable and the pathways are upregulated. *S. parasitica* was downregulated by copper sulfate treatment, and regulators in the energy supply were disturbed [59]. Valine, leucine and isoleucine can be broken down into glucose, which in turn produces ATP for energy [60,61]. Phenylalanine, tyrosine and tryptophan biosynthesis in the glycogenic pathway are transformed into fumaric and acetoacetic acids under the action of enzymes, the way by which they enter cellular energy metabolism. In addition, tyrosine can undergo phosphorylation, sulfation or nitration and therefore influence protein function [62,63]. These amino acid syntheses are closely related to energy. Following the copper sulfate treatment of *S. parasitica*, these amino acids were downregulated, blocking the synthesis of ATP, resulting in an ATP energy supply that was not smooth, and then led to cell death. The genes that were significantly enriched in pathway disorders were further analyzed. Glutamine synthetase (GS) is ubiquitous in all organisms and catalyzes the synthesis of glutamine from ammonia and glutamate using the energy released from the hydrolysis of ATP to ADP, and is also a key enzyme involved in nitrogen metabolism in organisms [64]. Nitrogen sources are important influential factors for cell growth and metabolism [65]. Amino acid metabolism is the main component of nitrogen metabolism. Arginine and nitrogen metabolism are involved in energy-related metabolism [66]. The downregulation of the relevant genes can lead to an insufficient energy supply of *S. parasitica* and the obstruction of related metabolic processes, which is consistent with the analysis conducted previously. Glucose-6-phosphate dehydrogenase (G6PD) is the main rate-limiting enzyme of the oxidative pentose phosphate pathway [67]. G6PD plays an essential role in the pentose phosphate shunt for reducing nicotinamide adenine dinucleotide phosphate (NADP) to nicotinamide adenine dinucleotide phosphate (NADPH) [68]. G6PD helps to maintain cellular redox homeostasis, whereas G6PD deficiency predisposes cells to increased oxidative stress. The G6PD three-dimensional (3D) structures show that each subunit binds one molecule of oxidized NADP, stabilizing the protein structure. In addition, the genetic results show that C-terminal (also termed A domains) and N-terminal domains (also termed C domains) are downregulated, which also leads to changes in some protein structures. In summary, it can be observed that the action of copper sulfate on *S. parasitica* causes its energy supply to be inhibited as well as the synthesis of related amino acids, the life activities of cells and cell information transmission.

Through the analysis of protein–protein interaction networks (PPIs), we selected five key genes (SPRG_08456, SPRG_03679, SPRG_04185, SPRG_10775 and SPRG_05141) involved in cellular and metabolic processes under the action of three antimicrobial agents. We came to understand their important roles in relation to metabolic pathways and their potential relationship with the mechanism of action of antimicrobial agents, whilst simultaneously discovering more ways to understand the way in which saprolegniasis is caused by *S. parasitica*. The aldehyde dehydrogenase family (ALDH) that is regulated by SPRG_08456 plays a significant role in the enzymatic detoxification of endogenous and exogenous aldehydes and in the formation of important molecules in cellular processes. The ALDH superfamily is represented in all three taxonomic domains (archaea, eubacteria and eukaryotes), suggesting a crucial role throughout evolutionary history. It has been demonstrated that members of this superfamily are used to metabolize aldehydes related to physiology and pathophysiology [69]. This ability prevents the accumulation of toxic aldehydes from endogenous production and exogenous exposure, thereby maintaining cellular homeostasis and normal functioning of the organism [70]. The ability of ALDH family members to metabolize active aldehydes represents a major potential cytoprotective mechanism, while accumulating evidence supports a role for ALDHs in regulating cell proliferation, differentiation and survival [71]. Therefore, the downregulation of the ALDH gene may threaten the survival of *S. parasitica*. The function of the SPRG_03679 gene was described in detail in the discussion of the control vs. bronopol group. The upregulation of ACADs regulated by SPRG_03679 may cause an oxidative stress response and serious damage to cell structures, which may lead to the gradual apoptosis of *S. parasitica*. This conclusion may be one of the reasons why *S. parasitica* stopped growing and even died under the action of chemical drugs in the antimicrobial test. Therefore, SPRG_03679 can be used as a potential target gene for further study. Coenzyme A (CoA) transferase catalyzes the reversible transfer of CoA between an existing acyl-CoA thioester and a free carboxylic acid. CoA transferases are a superfamily of proteins central to the metabolism of acetyl-CoA and other CoA thioesters. Class I CoA transferases are mainly involved in fatty acid metabolism, including those that transfer 3-keto acid, short-chain fatty acids and (E)-glutamic acid. Acetyl-CoA and succinyl-CoA are usually CoA donors. Class II CoA transferases catalyze the exchange of acetyl and citrate (or limonyl) groups on the thiols of the prosthesis groups to ensure the continuity of the catalytic cycle. Class III CoA transferases include enzymes involved in the metabolism of oxalate, carnitine, bile acids, toluene and other aromatic compounds, as well as succinyl-CoA: citrate CoA-transferase 2 fixation is involved in the 3-hydroxypropionate cycle of CO_2_ fixation [72]. The upregulation of CoA transferase under the action of antibacterial agents may lead to metabolic disorders that reduce antioxidant capacities, and ROS produced within the cells cannot be effectively removed to produce oxidative stress, resulting in reduced parasitic survival, which suggests that SPRG_10775 is a potential target gene for antimicrobials. SPRG_04185 and SPRG_05141 regulate two hypothesized proteins, which may be virulence genes in the genome of *S. parasitica* [73]. However, there is no further information on their role and function, so they are not suitable as target genes for antimicrobial screening.

## 5. Conclusions

In the current study, the inhibitory effects of metalaxyl, bronopol and copper sulfate on *S. parasitica* were evaluated, and copper sulfate possessed the best inhibitory effect, followed by bronopol. Through transcriptome experiments, it was observed that the three drugs inhibited the growth of *S. parasitica*, mainly by disrupting the amino acid metabolism, glutathione metabolism, arachidonic acid metabolism and glycerolipid metabolism pathways. The disorder of these pathways can affect multiple biological functions, including protein synthesis, oxidative stress, lipid metabolism and energy metabolism. The PPI network analysis revealed five important proteins networks corresponding to the genes SPRG_08456, SPRG_03679, SPRG_04185, SPRG_10775 and SPRG_05141, of which three key genes (SPRG_08456, SPRG_03679 and SPRG_10775) were screened as potential target genes of antimicrobials on *S. parasitica.* This will provide a scientific basis for the screening of other drugs for the prevention and control of saprolegniasis in the future and the healthy breeding of fish.

## Figures and Tables

**Table 1:** MIC and MBC values of metalaxyl, bronopol and copper sulfate against *Saprolegnia parasitica*.

Sample	MIC (mg/L)	MBC (mg/L)
Metalaxyl	5 mg/L	6 mg/L
Bronopol	4 mg/L	4 mg/L
Copper sulfate	2 mg/L	5 mg/L

Notes: Minimum inhibitory concentration (MIC) and minimum bactericidal concentration (MBC) are expressed as the mean concentration from three separate trials, all performed in triplicate.

## Data Availability

All available data are presented in the article.

## Supplementary Materials

The following supporting information can be downloaded at: https://www.mdpi.com/article/10.3390/genes13091524/s1. Table S1: Information of genes and specific primers for DEG validation; Table S2: Summary of reads in *S. parasitica* transcriptome sequencing; Table S3: Statistical results of trimmed reads mapping with reference genome; Table S4: DEGs of control vs. metalaxyl group assigned to GO categories and the terms are divided into three main GO categories; Table S5: DEGs of control vs. bronopol group assigned to GO categories and the terms are divided into three main GO categories; Table S6: DEGs of control vs. copper sulfate group assigned to GO categories and the terms are divided into three main GO categories; Table S7: DEGs of control vs. metalaxyl group assigned to KEGG pathway annotations; Table S8: DEGs of control vs. bronopol group assigned to KEGG pathway annotations; Table S9: DEGs of control vs. copper sulfate group assigned to KEGG pathway annotations; Table S10: KEGG-enriched analysis of DEGs in *S. parasitica* following metalaxyl, bronopol and copper sulfate treatments (*P_adj_* < 0.05); Table S11: The information of DEGs in enriched cysteine and methionine metabolism pathway in control vs. metalaxyl group; Table S12: The information of DEGs in enriched cysteine and methionine metabolism pathway in control vs. metalaxyl group; Table S13: The information of DEGs in enriched valine, leucine and isoleucine biosynthesis pathway in control vs. metalaxyl group; Table S14: The information of DEGs in enriched phenylalanine, tyrosine and tryptophan biosynthesis pathway in control vs. metalaxyl group; Table S15: The information of DEGs in enriched histidine metabolism pathway in control vs. metalaxyl group; Table S16: The information of DEGs in enriched valine, leucine and isoleucine degradation pathway in control vs. bronopol group; Table S17: The information of DEGs in enriched propanoate metabolism pathway in control vs. bronopol group; Table S18: The information of DEGs in enriched fatty acid degradation pathway in control vs. bronopol group; Table S19: The information of DEGs in enriched arginine biosynthesis pathway in control vs. copper sulfate; Table S20: The information of DEGs in enriched glutathione metabolism pathway in control vs. copper sulfate; Table S21: The information of DEGs in enriched valine, leucine and isoleucine biosynthesis pathway in control vs. copper sulfate; Table S22: The information of DEGs in enriched phenylalanine, tyrosine and tryptophan biosynthesis pathway in control vs. copper sulfate; Figure S1: Cysteine and methionine metabolism pathway enriched by significantly different gene in control vs. metalaxyl group; Figure S2: Glutathione metabolism pathway enriched by significantly different gene in control vs. metalaxyl group; Figure S3: Valine, leucine and isoleucine biosynthesis pathway enriched by significantly different gene in control vs. metalaxyl group; Figure S4: Phenylalanine, tyrosine and tryptophan biosynthesis pathway enriched by significantly different gene in control vs. metalaxyl group; Figure S5: Histidine metabolism pathway enriched by significantly different gene in control vs. metalaxyl group; Figure S6: Valine, leucine and isoleucine degradation pathway enriched by significantly different gene in control vs. bronopol group; Figure S7: Propanoate metabolism pathway enriched by significantly different gene in control vs. bronopol group; Figure S8: Fatty acid degradation pathway enriched by significantly different gene in control vs. bronopol group; Figure S9: Arginine biosynthesis pathway enriched by significantly different gene in control vs. copper sulfate; Figure S10: Glutathione metabolism pathway enriched by significantly different gene in control vs. copper sulfate; Figure S11: Valine, leucine and isoleucine biosynthesis pathway enriched by significantly different gene in control vs. copper sulfate; Figure S12: Phenylalanine, tyrosine and tryptophan biosynthesis pathway enriched by significantly different gene in control vs. copper sulfate.
