# Supplementary material for: Characterizing the Mechanisms of Metalaxyl, Bronopol and Copper Sulfate against Saprolegnia parasitica Using Modern Transcriptomics
Source: Genes (Basel). 2022 Aug 25;13(9):1524. doi: 10.3390/genes13091524 (PMC9498376; doi:10.3390/genes13091524)
Supplement: Supplementary file 1 [file genes-13-01524-s001.zip › genes-1875845-supplementary.pdf]

Table S1. Information of genes and specific primers for DEGs validation.

Gene name	Gene ID	Sequence (5'-3')	Product length
SPRG_00366	gene372_F	CTCGGCATGGATGGTGTCT	104
	gene372_R	GCGTCCTTGTAGTGCGTGA	
SPRG_06183	gene6929_F	CTTTGCCGTGTCCATCGT	133
	gene6929_R	AAGGCTCGTGCCCTCGTT	
SPRG_02474	gene2966_F	TCAATTCCAATGACCCAAAC	192
	gene2966_R	CACTTCGTGCATAAAGTCCAG	
SPRG_11531	gene12591_F	TATGGGCTACGGCCAACC	146
	gene12591_R	CGGCGATTTCGTCCTCCT	
SPRG_03679	gene4196_F	TTGGCGAGGTAGATAGTATTGC	185
	gene4196_R	TGATGGCGGTCAAAGAGGT	
SPRG_08710	gene9963_F	CGTTTGCGGTCATCCTCTT	118
	gene9963_R	GGCGTCTCAATGTCGTGGT	
SpTub-b	SpTub_bF	AGACGGGTGCTGGTAACAAC	136
	SpTub_bR	AGCGAGTGCGTAATCTGGAAA	

Table S2. Summary of reads in *S. parasitica* transcriptome sequencing.

Groups	Sample	Raw reads	Clean reads	Error rate (%)	Q30 (%)	GC content (%)
Control	Control_1	54,827,654	54,405,806	0.0226	96.74	61.42
	Control_2	54,783,272	54,309,506	0.0226	96.73	61.23
	Control_3	52,796,972	52,307,606	0.0226	96.73	61.05
Metalaxyl	Metalaxyl_1	60,395,304	59,937,938	0.0227	96.54	62.11
	Metalaxyl_2	56,350,680	55,795,310	0.0226	96.75	61.35
	Metalaxyl_3	51,589,090	51,066,664	0.0227	96.63	61.46
Bronopol	Bronopol_1	53,299,518	52,721,412	0.0226	96.67	61.83
	Bronopol_2	45,096,636	44,746,740	0.0255	93.71	61.98
	Bronopol_2	51,634,630	51,161,658	0.0225	96.76	62.07
Copper Sulfate	Copper_Sulfate_1	54,995,012	54,539,128	0.0226	96.66	62.04
	Copper_Sulfate_2	51,933,312	51,589,302	0.0255	93.73	62.04
	Copper_Sulfate_3	51,446,352	50,955,844	0.0226	96.73	61.98

Notes: Raw reads is the total number of original offline sequences; Clean reads is the sequence number after quality control; Error rate is the sequencing Error rate; Clean Q30 bases rate is the proportion of bases with the mass value greater than 30 in clean reads sequence; GC content (%) is the percentage of the total number of bases g and C in the total number of bases.

Table S3. Statistical results of trimmed reads mapping with reference genome.

Sample	Total reads	Total mapped	Multiple mapped	Uniquely mapped
Control_1	54,405,806	47,655,973(87.59%)	3,913,760(7.19%)	43,742,213(80.4%)
Control_2	54,309,506	47,729,555(87.88%)	4,567,903(8.41%)	43,161,652(79.47%)
Control_3	52,307,606	46,241,343(88.4%)	4,862,002(9.3%)	41,379,341(79.11%)
Metalaxyl_1	59,937,938	51,250,485(85.51%)	2,585,051(4.31%)	48,665,434(81.19%)

Metalaxyl_2	55,795,310	48,976,950(87.78%)	4,214,145(7.55%)	44,762,805(80.23%)
Metalaxyl_3	51,066,664	44,356,444(86.86%)	3,723,860(7.29%)	40,632,584(79.57%)
Bronopol_1	52,721,412	45,550,122(86.4%)	2,811,896(5.33%)	42,738,226(81.06%)
Bronopol_2	44,746,740	37,910,306(84.72%)	2,140,296(4.78%)	35,770,010(79.94%)
Bronopol_3	51,161,658	43,726,944(85.47%)	2,383,506(4.66%)	41,343,438(80.81%)
Copper_Sulfate_1	54,539,128	47,080,882(86.32%)	2,443,511(4.48%)	44,637,371(81.84%)
Copper_Sulfate_2	51,589,302	44,171,759(85.62%)	2,496,801(4.84%)	41,674,958(80.78%)
Copper_Sulfate_3	50,955,844	44,181,274(86.71%)	2,646,346(5.19%)	41,534,928(81.51%)

Notes: Total reads is the sequence number after quality control; Total mapped is the number of clean reads that can be mapped on the genome; Unique mapped is clean reads that have a unique position on the reference sequence.

Table S4. DEGs of control vs metalaxyl group were assigned to GO categories and the terms were summarized into three main GO categories.

GO ID	Description	Term Type	Number	Percent	Up-regulated	Down-regulated
GO:0032501	multicellular organismal process	BP	1	1/3187	1	0
GO:0065007	biological regulation	BP	139	139/3187	74	65
GO:0051704	multi-organism process	BP	19	19/3187	13	6
GO:0022414	reproductive process	BP	4	4/3187	2	2
GO:0040011	locomotion	BP	2	2/3187	1	1
GO:0050896	response to stimulus	BP	54	54/3187	20	34
GO:0009987	cellular process	BP	635	635/3187	292	343
GO:0098754	detoxification	BP	2	2/3187	0	2
GO:0022610	biological adhesion	BP	1	1/3187	1	0
GO:0015976	carbon utilization	BP	2	2/3187	0	2
GO:0001906	cell killing	BP	2	2/3187	2	0
GO:0051179	localization	BP	125	125/3187	89	36
GO:0032502	developmental process	BP	3	3/3187	2	1
GO:0071840	cellular component organization or biogenesis	BP	69	69/3187	32	37
GO:0008152	metabolic process	BP	634	634/3187	300	334
GO:0044425	membrane part	CC	1286	1286/3187	799	487
GO:0005623	cell	CC	41	41/3187	23	18
GO:0043226	organelle	CC	203	203/3187	106	97
GO:0044422	organelle part	CC	144	144/3187	71	73
GO:0032991	protein-containing complex	CC	147	147/3187	71	76
GO:0044464	cell part	CC	400	400/3187	213	187
GO:0016020	membrane	CC	39	39/3187	29	10
GO:0099080	supramolecular complex	CC	20	20/3187	10	10
GO:0031974	membrane-enclosed lumen	CC	5	5/3187	1	4
GO:0005576	extracellular region	CC	61	61/3187	37	24
GO:0140312	cargo adaptor activity	MF	1	1/3187	1	0

GO:0005215	transporter activity	MF	260	260/3187	165	95
GO:0005488	binding	MF	1208	1208/3187	644	564
GO:0003824	catalytic activity	MF	1355	1355/3187	671	684
GO:0005198	structural molecule activity	MF	30	30/3187	17	13
GO:0098772	molecular function regulator	MF	26	26/3187	13	13
GO:0045735	nutrient reservoir activity	MF	1	1/3187	1	0
GO:0016209	antioxidant activity	MF	9	9/3187	3	6
GO:0045182	translation regulator activity	MF	20	20/3187	12	8
GO:0060089	molecular transducer activity	MF	4	4/3187	3	1
GO:0140110	transcription regulator activity	MF	28	28/3187	10	18

Notes: GO ID indicates the identification number of each enriched GO-term; Description shows the secondary classification of hierarchical GO terms; Term type shows the primary classification of GO terms, including biological processes (BP), cellular components (CC), and molecular functions (MF); Number shows the number of genes annotated to the GO secondary classification function; Percent shows the percentage of genes annotated with the GO secondary classification function; Up-regulated and down-regulated show the number of up-regulated/ down-regulated genes in corresponding GO terms.

Table S5. DEGs of control vs bronopol group were assigned to GO categories and the terms were summarized into three main GO categories.

GO ID	Description	Term Type	Number	Percent	Up-regulated	Down-regulated
GO:0065007	biological regulation	BP	40	40/1042	31	9
GO:0051704	multi-organism process	BP	2	2/1042	1	1
GO:0022414	reproductive process	BP	1	1/1042	1	0
GO:0050896	response to stimulus	BP	9	9/1042	4	5
GO:0009987	cellular process	BP	176	176/1042	125	51
GO:0051179	localization	BP	38	38/1042	35	3
GO:0032502	developmental process	BP	1	1/1042	1	0
GO:0071840	cellular component organization or biogenesis	BP	16	16/1042	9	7
GO:0008152	metabolic process	BP	186	186/1042	132	54
GO:0044425	membrane part	CC	446	446/1042	314	132
GO:0005623	cell	CC	13	13/1042	10	3
GO:0043226	organelle	CC	52	52/1042	39	13
GO:0044422	organelle part	CC	38	38/1042	29	9
GO:0032991	protein-containing complex	CC	33	33/1042	24	9
GO:0044464	cell part	CC	112	112/1042	86	26
GO:0016020	membrane	CC	14	14/1042	12	2
GO:0099080	supramolecular complex	CC	7	7/1042	5	2
GO:0005576	extracellular region	CC	20	20/1042	16	4
GO:0140312	cargo adaptor activity	MF	1	1/1042	1	0
GO:0005215	transporter activity	MF	94	94/1042	73	21

GO:0005488	binding	MF	358	358/1042	248	110
GO:0003824	catalytic activity	MF	427	427/1042	301	126
GO:0005198	structural molecule activity	MF	5	5/1042	5	0
GO:0098772	molecular function regulator	MF	11	11/1042	7	4
GO:0140110	transcription regulator activity	MF	9	9/1042	4	5

Notes: The head of the table has the same meaning as table S4.

Table S6. DEGs of control vs copper sulfate group were assigned to GO categories and the terms were summarized into three main GO categories.

GO ID	Description	Term Type	Number	Percent	Up-regulated	Down-regulated
GO:0032501	multicellular organismal process	BP	2	2/4279	1	1
GO:0065007	biological regulation	BP	202	202/4279	82	120
GO:0051704	multi-organism process	BP	26	26/4279	15	11
GO:0022414	reproductive process	BP	6	6/4279	2	4
GO:0040011	locomotion	BP	2	2/4279	1	1
GO:0050896	response to stimulus	BP	79	79/4279	18	61
GO:0009987	cellular process	BP	909	909/4279	348	561
GO:0098754	detoxification	BP	2	2/4279	0	2
GO:0022610	biological adhesion	BP	1	1/4279	1	0
GO:0015976	carbon utilization	BP	2	2/4279	0	2
GO:0001906	cell killing	BP	2	2/4279	2	0
GO:0051179	localization	BP	178	178/4279	99	79
GO:0032502	developmental process	BP	5	5/4279	3	2
GO:0071840	cellular component organization or biogenesis	BP	117	117/4279	38	79
GO:0008152	metabolic process	BP	845	845/4279	338	507
GO:0044425	membrane part	CC	1643	1643/4279	913	730
GO:0005623	cell	CC	56	56/4279	24	32
GO:0043226	organelle	CC	286	286/4279	122	164
GO:0044422	organelle part	CC	222	222/4279	89	133
GO:0032991	protein-containing complex	CC	249	249/4279	95	154
GO:0044464	cell part	CC	591	591/4279	250	341
GO:0016020	membrane	CC	56	56/4279	32	24
GO:0099080	supramolecular complex	CC	29	29/4279	14	15
GO:0031974	membrane-enclosed lumen	CC	6	6/4279	1	5
GO:0005576	extracellular region	CC	78	78/4279	46	32
GO:0140312	cargo adaptor activity	MF	1	1/4279	1	0
GO:0005215	transporter activity	MF	314	314/4279	175	139
GO:0005488	binding	MF	1678	1678/4279	772	906
GO:0003824	catalytic activity	MF	1806	1806/4279	782	1024

GO:0140104	molecular carrier activity	MF	1	1/4279	0	1
GO:0005198	structural molecule activity	MF	46	46/4279	21	25
GO:0098772	molecular function regulator	MF	44	44/4279	13	31
GO:0045735	nutrient reservoir activity	MF	1	1/4279	1	0
GO:0016209	antioxidant activity	MF	17	17/4279	5	12
GO:0045182	translation regulator activity	MF	24	24/4279	11	13
GO:0060089	molecular transducer activity	MF	7	7/4279	3	4
GO:0140110	transcription regulator activity	MF	29	29/4279	10	19
GO:0140299	small molecule sensor activity	MF	1	1/4279	0	1

Notes: The head of the table has the same meaning as table S4.

Table S7. DEGs of control vs metalaxyl group were assigned to KEGG pathway annotations.

First category	Second category	Pathway ID	DEGs number
Metabolism	Amino acid metabolism	map00310 map00260 map00250 map00300 map00380 map00270 map00360 map00280 map00350 map00290 map00220 map00340 map00330 map00400	188
	Carbohydrate metabolism	map00650 map00040 map00562 map00053 map00052 map00020 map00500 map00030 map00620 map00630 map00520 map00010 map00640	126
	Lipid metabolism	map00061 map00073 map00072 map00071 map00062 map00565 map00564 map00561 map00600 map00100 map00590 map00592 map01040	121
	Metabolism of cofactors and vitamins	map00790 map00860 map00780 map00670 map00770 map00760 map00750 map00130 map00740 map00730	58
	Nucleotide metabolism	map00230 map00240	47
	Metabolism of other amino acids	map00410 map00460 map00450 map00430 map00480	45
	Energy metabolism	map00710 map00910 map00190 map00920	21
	Glycan biosynthesis and metabolism	map00563 map00603 map00604 map00511 map00513 map00514 map00531	14
	Biosynthesis of other secondary metabolites	map00261 map00524	6
	Metabolism of terpenoids and polyketides	map00900	4
	Endocrine and metabolic disease	map04933	4
Genetic Information Processing	Folding, sorting and degradation	map04130 map03060 map04120 map03018 map04141 map03050	52
	Translation	map00970 map03008 map03010 map03015 map03013	42
	Replication and repair	map03410 map03430 map03420 map03030 map03450 map03440	35
	Transcription	map03040 map03020	9
Cellular Processes	Transport and catabolism	map04136 map04146 map04145 map04144	50

**Human Diseases**	Infectious disease: parasitic	map05140 map05146 map05145	49
	Endocrine and metabolic disease	map04933	4
**Environmental Information Processing**	Membrane transport	map02010	17
	Signal transduction	map04070	15

Table S8. DEGs of control vs bronopol group were assigned to KEGG pathway annotations.

Frist category	Second category	Pathway ID	DEGs number
**Metabolism**	Lipid metabolism	map00061 map00100 map00590 map00591 map00062 map01040 map00071 map00565 map00564 map00561 map00072 map00600 map00592	74
	Amino acid metabolism	map00310 map00270 map00330 map00250 map00350 map00300 map00340 map00360 map00260 map00400 map00380 map00280	67
	Carbohydrate metabolism	map00040 map00500 map00630 map00650 map00520 map00562 map00053 map00010 map00640 map00620 map00040 map00500	52
	Nucleotide metabolism	map00230 map00240	24
	Metabolism of cofactors and vitamins	map00790 map00750 map00860 map00670 map00740 map00130 map00760 map00730	20
	Metabolism of other amino acids	map00410 map00480 map00430	16
	Energy metabolism	map00710 map00190 map00910	7
	Glycan biosynthesis and metabolism	map00531 map00511 map00513 map00514	4
	Metabolism of terpenoids and polyketides	map00900	1
	Biosynthesis of other secondary metabolites	map00232	1
**Cellular Processes**	Transport and catabolism	map04136 map04145 map04144 map04146	16
**Human Diseases**	Infectious disease: parasitic	map05140 map05145 map05146	14
	Endocrine and metabolic disease	map04933	2
**Genetic Information Processing**	Folding, sorting and degradation	map03060 map04130 map04120 map04141	8
	Translation	map03010 map03015	3
	Transcription	map03040	1
	Replication and repair	map03410	1
**Environmental Information Processing**	Signal transduction	map04070	4
	Membrane transport	map02010	1

Table S9. DEGs of control vs copper sulfate group were assigned to KEGG pathway annotations.

Frist category	Second category	Pathway ID	DEGs number
Metabolism	Amino acid metabolism	map00310 map00260 map00250 map00300 map00380 map00270 map00360 map00280 map00350 map00290 map00220 map00340 map00330 map00400	203
	Carbohydrate metabolism	map00650 map00040 map00562 map00051 map00053 map00052 map00020 map00500 map00030 map00620 map00630 map00520 map00010 map00640	146
	Lipid metabolism	map00061 map00073 map00072 map00071 map00062 map00565 map00564 map00561 map00600 map00100 map00590 map00591 map00592 map01040	144
	Metabolism of cofactors and vitamins	map00790 map00860 map00780 map00670 map00770 map00760 map00750 map00130 map00740 map00730	71
	Metabolism of other amino acids	map00410 map00460 map00472 map00450 map00430 map00480	56
	Nucleotide metabolism	map00230 map00240	51
	Energy metabolism	map00710 map00910 map00190 map00920	33
	Glycan biosynthesis and metabolism	map00563 map00603 map00511 map00513 map00514 map00604 map00531	18
	Biosynthesis of other secondary metabolites	map00261 map00232 map00524	9
	Metabolism of terpenoids and polyketides	map00900	4
Genetic Information Processing	Replication and repair	map03410 map03430 map03420 map03030 map03450 map03440	88
	Folding, sorting and degradation	map04130 map03060 map04120 map04122 map03018 map04141 map03050	85
	Translation	map00970 map03008 map03010 map03015 map03013	54
	Transcription	map03040 map03020	15
Cellular Processes	Transport and catabolism	map04136 map04146 map04145 map04144	81
Human Diseases	Infectious disease: parasitic	map05140 map05143 map05146 map05145	76
	Endocrine and metabolic disease	map04933	12
Environmental Information Processing	Signal transduction	map04070	25
	Membrane transport	map02010	25

Table S10. KEGG enriched analysis of DEGs in *S. parasitica* following metalaxyl, bronopol and copper sulfate treatment ($P_{adj} < 0.05$).

Groups	Name	Map	Genes number	Padjust	First Category	Second Category
control vs metalaxyl group	Cysteine and methionine metabolism	map00270	24	0.000106	Metabolism	Amino acid metabolism
control vs metalaxyl group	Glutathione metabolism	map00480	27	0.000130	Metabolism	Metabolism of other amino acids
control vs metalaxyl group	Valine, leucine and isoleucine biosynthesis	map00290	10	0.000159	Metabolism	Amino acid metabolism
control vs metalaxyl group	Phenylalanine, tyrosine and tryptophan biosynthesis	map00400	13	0.000203	Metabolism	Amino acid metabolism
control vs metalaxyl group	Histidine metabolism	map00340	13	0.000914	Metabolism	Amino acid metabolism
control vs metalaxyl group	Arginine biosynthesis	map00220	14	0.001029	Metabolism	Amino acid metabolism
control vs metalaxyl group	Fatty acid degradation	map00071	27	0.001926	Metabolism	Lipid metabolism
control vs metalaxyl group	Arachidonic acid metabolism	map00590	19	0.002031	Metabolism	Lipid metabolism
control vs metalaxyl group	Tryptophan metabolism	map00380	14	0.003541	Metabolism	Amino acid metabolism
control vs metalaxyl group	Glycerolipid metabolism	map00561	20	0.003789	Metabolism	Lipid metabolism
control vs metalaxyl group	Valine, leucine and isoleucine degradation	map00280	21	0.003914	Metabolism	Amino acid metabolism
control vs metalaxyl group	Pantothenate and CoA biosynthesis	map00770	10	0.011656	Metabolism	Metabolism of cofactors and vitamins
control vs metalaxyl group	Tyrosine metabolism	map00350	14	0.012323	Metabolism	Amino acid metabolism
control vs metalaxyl group	Purine metabolism	map00230	31	0.012413	Metabolism	Nucleotide metabolism
control vs metalaxyl group	Lysine biosynthesis	map00300	7	0.041937	Metabolism	Amino acid metabolism
control vs metalaxyl group	Glycine, serine and threonine metabolism	map00260	16	0.043679	Metabolism	Amino acid metabolism
control vs metalaxyl group	Starch and sucrose metabolism	map00500	24	0.044995	Metabolism	Carbohydrate metabolism
control vs bronopol group	Valine, leucine and isoleucine degradation	map00280	19	0.000001	Metabolism	Amino acid metabolism
control vs bronopol group	Propanoate metabolism	map00640	11	0.000040	Metabolism	Carbohydrate metabolism
control vs bronopol group	Fatty acid degradation	map00071	16	0.000040	Metabolism	Lipid metabolism
control vs bronopol group	Tyrosine metabolism	map00350	9	0.001134	Metabolism	Amino acid metabolism
control vs bronopol group	Glycerolipid metabolism	map00561	11	0.001150	Metabolism	Lipid metabolism
control vs bronopol group	Arachidonic acid metabolism	map00590	10	0.001302	Metabolism	Lipid metabolism
control vs bronopol group	Tryptophan metabolism	map00380	8	0.001446	Metabolism	Amino acid metabolism
control vs bronopol group	Pyrimidine metabolism	map00240	10	0.004535	Metabolism	Nucleotide metabolism
control vs bronopol group	Lysine degradation	map00310	9	0.007786	Metabolism	Amino acid metabolism
control vs bronopol group	Purine metabolism	map00230	14	0.008249	Metabolism	Nucleotide metabolism
control vs bronopol group	Steroid biosynthesis	map00100	6	0.010920	Metabolism	Lipid metabolism
control vs bronopol group	beta-Alanine metabolism	map00410	6	0.013453	Metabolism	Metabolism of other amino acids
control vs bronopol group	Nicotinate and nicotinamide metabolism	map00760	7	0.014400	Metabolism	Metabolism of cofactors and vitamins
control vs bronopol group	Phenylalanine metabolism	map00360	4	0.023083	Metabolism	Amino acid metabolism

	Pentose and glucuronate interconversions	map00040	4	0.027677	Metabolism	Carbohydrate metabolism
	Butanoate metabolism	map00650	5	0.028397	Metabolism	Carbohydrate metabolism
	Glutathione metabolism	map00480	9	0.029118	Metabolism	Metabolism of other amino acids
	Fatty acid elongation	map00062	4	0.043950	Metabolism	Lipid metabolism
	Glycerophospholipid metabolism	map00564	9	0.044535	Metabolism	Lipid metabolism
control vs copper sulfate	Arginine biosynthesis	map00220	20	0.000013	Metabolism	Amino acid metabolism
	Glutathione metabolism	map00480	33	0.000169	Metabolism	Metabolism of other amino acids
	Valine, leucine and isoleucine biosynthesis	map00290	11	0.000291	Metabolism	Amino acid metabolism
	Phenylalanine, tyrosine and tryptophan biosynthesis	map00400	14	0.000612	Metabolism	Amino acid metabolism
	Cysteine and methionine metabolism	map00270	25	0.001787	Metabolism	Amino acid metabolism
	Glycerolipid metabolism	map00561	25	0.004120	Metabolism	Lipid metabolism
	Histidine metabolism	map00340	14	0.004394	Metabolism	Amino acid metabolism
	Lysine biosynthesis	map00300	10	0.005294	Metabolism	Amino acid metabolism
	Arachidonic acid metabolism	map00590	22	0.005342	Metabolism	Lipid metabolism
	Nitrogen metabolism	map00910	12	0.012155	Metabolism	Energy metabolism
	Homologous recombination	map03440	21	0.025520	Genetic Information Processing	Replication and repair
	Alanine, aspartate and glutamate metabolism	map00250	18	0.032321	Metabolism	Amino acid metabolism
	Pantothenate and CoA biosynthesis	map00770	11	0.036134	Metabolism	Metabolism of cofactors and vitamins
	alpha-Linolenic acid metabolism	map00592	7	0.044645	Metabolism	Lipid metabolism

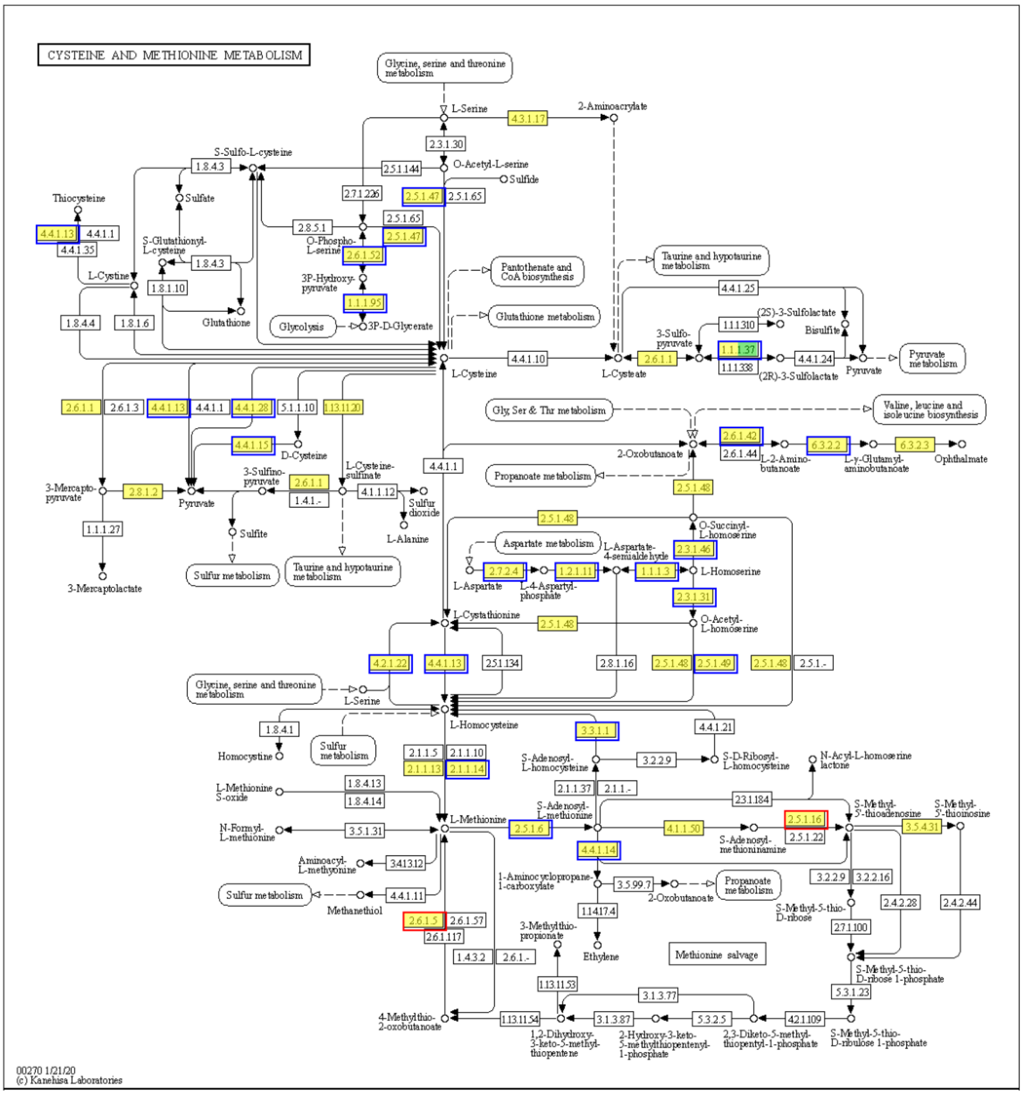

Figure S1. Cysteine and methionine metabolism pathway enriched by significantly different gene in control vs metalaxyl group.

Notes: Blue represents downregulated genes; Red represents upregulated genes.

Table S11. The information of DEGs in enriched cysteine and methionine metabolism pathway in control vs metalaxyl group.

Number	Gene ID	gene name	KO name	Pfam Description	log2FC
1	gene7273	SPRG_06475	metC	Cys/Met metabolism PLP-dependent enzyme;Aminotransferase class-V;DegT/DnrJ/EryC1/StrS aminotransferase family;Aminotransferase class I and II	-2.22
2	gene7990	SPRG_07198	dcyD	Pyridoxal-phosphate dependent enzyme	-1.08
3	gene2285	SPRG_01810	metY	Cys/Met metabolism PLP-dependent enzyme;Aminotransferase class I and II;DegT/DnrJ/EryC1/StrS aminotransferase family;Aminotransferase class-V;Beta-eliminating lyase	-2.96

4	gene4524	SPRG_03997	DNAH;ACS;ACS1_2_6	Aminotransferase class I and II	-2.16
5	gene9590	SPRG_08341	asd	Semialdehyde dehydrogenase, dimerisation domain;Semialdehyde dehydrogenase, NAD binding domain	-1.24
6	gene6907	SPRG_06162	cysK	Cys/Met metabolism PLP-dependent enzyme	-2.31
7	gene7454	SPRG_06652	metX	alpha/beta hydrolase fold;Alpha/beta hydrolase family	-1.29
8	gene4084	SPRG_03567	GCLC	Glutamate-cysteine ligase	-2.20
9	gene2383	SPRG_01903	E2.6.1.42, ilvE	Amino-transferase class IV	-2.99
10	gene8138	SPRG_07349	metE	Cobalamin-independent synthase, Catalytic domain;Cobalamin-independent synthase, N-terminal domain	-5.25
11	gene19974	SPRG_18377	metX	alpha/beta hydrolase fold;Alpha/beta hydrolase family	-1.50
12	gene11112	SPRG_09635	E3.3.1.1, ahcY	S-adenosyl-L-homocysteine hydrolase;S-adenosyl-L-homocysteine hydrolase, NAD binding domain;D-isomer specific 2-hydroxyacid dehydrogenase, NAD binding domain;Acetohydroxy acid isomeroreductase, NADPH-binding domain	-1.43
13	gene2941	SPRG_02449	thrA	Amino acid kinase family;Homoserine dehydrogenase;Homoserine dehydrogenase, NAD binding domain	-1.98
14	gene18661	SPRG_17078	E3.3.1.1, ahcY	S-adenosyl-L-homocysteine hydrolase;S-adenosyl-L-homocysteine hydrolase, NAD binding domain	-1.11
15	gene10672	SPRG_09443	lysAC	Amino acid kinase family	-1.51
16	gene4889	SPRG_04306	serC, PSAT1	D-isomer specific 2-hydroxyacid dehydrogenase, NAD binding domain;D-isomer specific 2-hydroxyacid dehydrogenase, catalytic domain;ACT domain;NAD binding domain of 6-phosphogluconate dehydrogenase	-1.11
17	gene2863	SPRG_02373	serA, PHGDH	D-isomer specific 2-hydroxyacid dehydrogenase, NAD binding domain;D-isomer specific 2-hydroxyacid dehydrogenase, catalytic domain;NAD binding domain of 6-phosphogluconate dehydrogenase;ACT domain	-1.07
18	gene17818	SPRG_16389	speE, SRM, SPE3	none	1.11
19	gene10556	SPRG_09101	MDH1	lactate/malate dehydrogenase, alpha/beta C-terminal domain;lactate/malate dehydrogenase, NAD binding domain	-1.14
20	gene3757	SPRG_03249	metK	S-adenosylmethionine synthetase, C-terminal domain;S-adenosylmethionine synthetase, central domain;S-adenosylmethionine synthetase, N-terminal domain	-1.60
21	gene7858	SPRG_06771	TAT	Aminotransferase class I and II;Cys/Met metabolism PLP-dependent enzyme	3.52
22	gene11693	SPRG_09898	cysK	Pyridoxal-phosphate dependent enzyme	-1.52
23	gene11396	SPRG_10298	CBS	Pyridoxal-phosphate dependent enzyme;CBS domain	-1.31
24	gene1236	SPRG_01114	LCD	none	-1.83

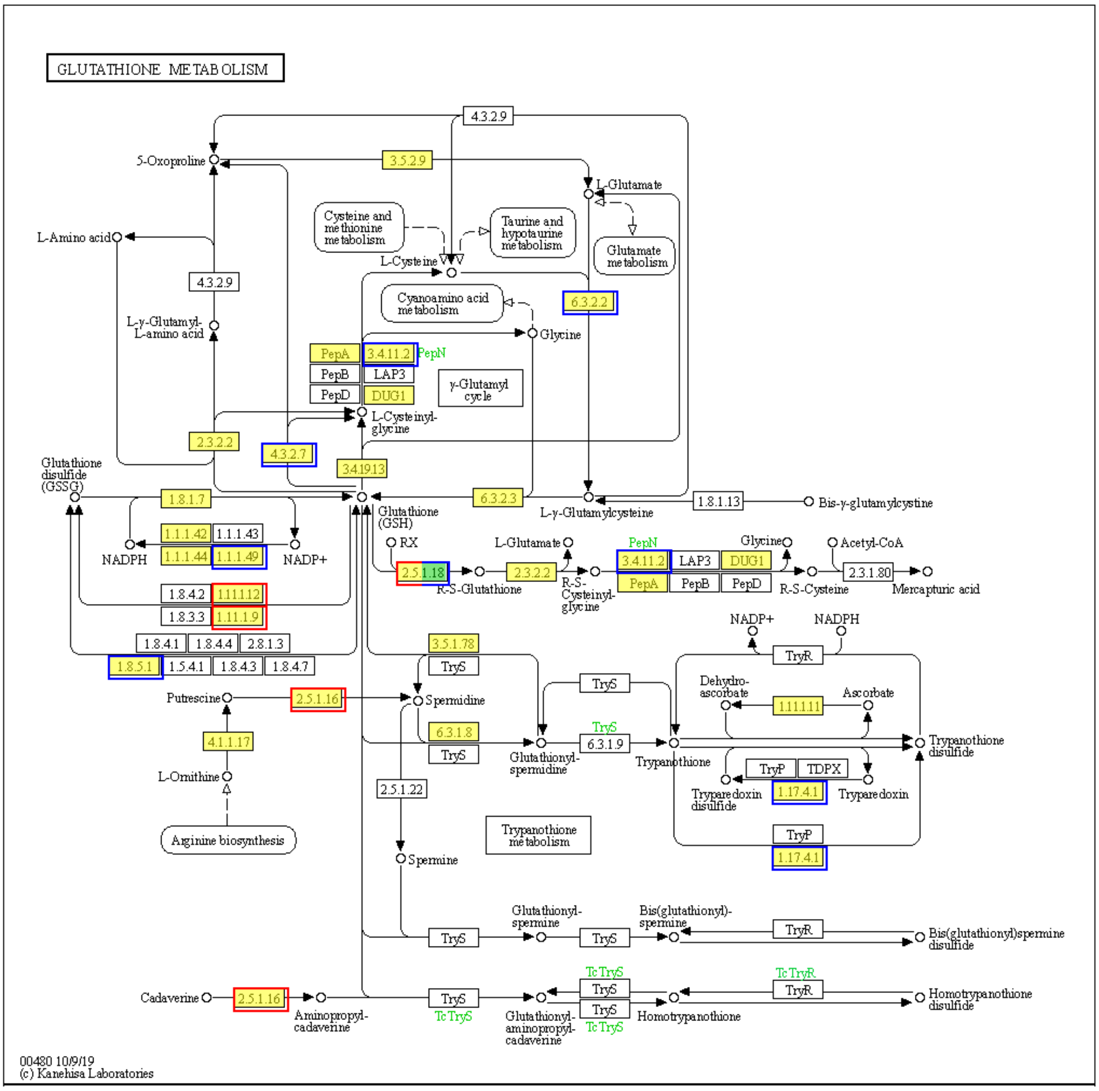

Figure S2. Glutathione metabolism pathway enriched by significantly different gene in control vs metalaxyl group.

Notes: Blue represents downregulated genes; Red represents upregulated genes.

Table S12. The information of DEGs in enriched cysteine and methionine metabolism pathway in control vs metalaxyl group.

Number	Gene ID	gene name	KO name	Pfam Description	log2FC
1	gene14098	SPRG_12364	K21888	Glutathione S-transferase, C-terminal domain;Glutathione S-transferase, N-terminal domain;Glutathione S-transferase, N-terminal domain;Glutathione S-transferase, C-terminal domain;Glutathione S-transferase, N-terminal domain;Glutathione S-transferase, C-terminal domain	-3.10
2	gene514	SPRG_18820	pif;K00799	MAPEG family	-3.33
3	gene10469	SPRG_09358	K04097	Glutathione S-transferase, C-terminal domain;Glutathione S-transferase, N-terminal domain;Glutathione S-transferase, C-terminal domain	-2.93
4	gene14826	SPRG_12917	K06207;K07232	ChaC-like protein;Gamma-glutamyl cyclotransferase, AIG2-like;AIG2-like family	-1.03
5	gene7600	SPRG_06230	K04097	Glutathione S-transferase, C-terminal domain	6.97
6	gene6929	SPRG_06183	K00799	MAPEG family	-1.19
7	gene6928	SPRG_06182	K00799	MAPEG family	2.55
8	gene14539	SPRG_12421	K00036	Glucose-6-phosphate dehydrogenase, C-terminal domain;Glucose-6-phosphate dehydrogenase, NAD binding domain	-1.72
9	gene7868	SPRG_20281	K04097	Glutathione S-transferase, C-terminal domain;Glutathione S-transferase, N-terminal domain;Glutathione S-transferase, C-terminal domain	1.52
10	gene17946	SPRG_16543	K04097	Glutathione S-transferase, C-terminal domain;Glutathione S-transferase, C-terminal domain;Glutathione S-transferase, N-terminal domain;Glutathione S-transferase, C-terminal domain	1.71
11	gene4084	SPRG_03567	K11204	Glutamate-cysteine ligase	-2.20
12	gene15352	SPRG_12960	K04097	Glutathione S-transferase, C-terminal domain;Glutathione S-transferase, C-terminal domain;Glutathione S-transferase, N-terminal domain	1.91
13	gene4646	SPRG_04116	K10807	Ribonucleotide reductase, barrel domain;Ribonucleotide reductase, all-alpha domain;ATP cone domain	-1.42
14	gene9670	SPRG_08418	K00799	MAPEG family	-2.01
15	gene16910	SPRG_15022	K04097;K06911	Glutathione S-transferase, C-terminal domain	-1.10
16	gene13584	SPRG_11344	K04097;K06911	Glutathione S-transferase, C-terminal domain;Glutathione S-transferase, N-terminal domain;Glutathione S-transferase, C-terminal domain	6.16
17	gene18969	SPRG_17314	K00799	MAPEG family	-1.76
18	gene7599	SPRG_22185	K04097;K06911	Glutathione S-transferase, C-terminal domain;Glutathione S-transferase, C-terminal domain;Glutathione S-transferase, C-terminal domain	5.82
19	gene4590	SPRG_04061	K01256	Peptidase family M1 domain;Domain of unknown function;Peptidase M1 N-terminal domain;Domain of unknown function	-1.71

20	gene17818	SPRG_16389	K00797	none	1.11
21	gene9072	SPRG_08082	K00432;K05361	Glutathione peroxidase	1.61
22	gene15668	SPRG_14435	K10808	Ribonucleotide reductase, small chain	-1.51
23	gene13585	SPRG_11345	K04097;K06911	Glutathione S-transferase, C-terminal domain;Glutathione S-transferase, N-terminal domain	6.78
24	gene17654	SPRG_16152	K21888	Glutathione S-transferase, C-terminal domain;Glutathione S-transferase, N-terminal domain;Glutathione S-transferase, N-terminal domain;Glutathione S-transferase, C-terminal domain;Glutathione S-transferase, N-terminal domain;Glutathione S-transferase, C-terminal domain	-3.18
25	gene19865	SPRG_18265	K10808	Ribonucleotide reductase, small chain	-1.39
26	gene11247	SPRG_10256	K00799	Glutathione S-transferase, N-terminal domain;Glutathione S-transferase, N-terminal domain;Glutathione S-transferase, N-terminal domain;Glutathione S-transferase, C-terminal domain;Glutathione S-transferase, C-terminal domain	-4.15
27	gene17653	SPRG_16151	K21888	Glutathione S-transferase, C-terminal domain;Glutathione S-transferase, N-terminal domain;Glutathione S-transferase, C-terminal domain;Glutathione S-transferase, N-terminal domain;Glutathione S-transferase, N-terminal domain;Glutathione S-transferase, C-terminal domain	-2.59

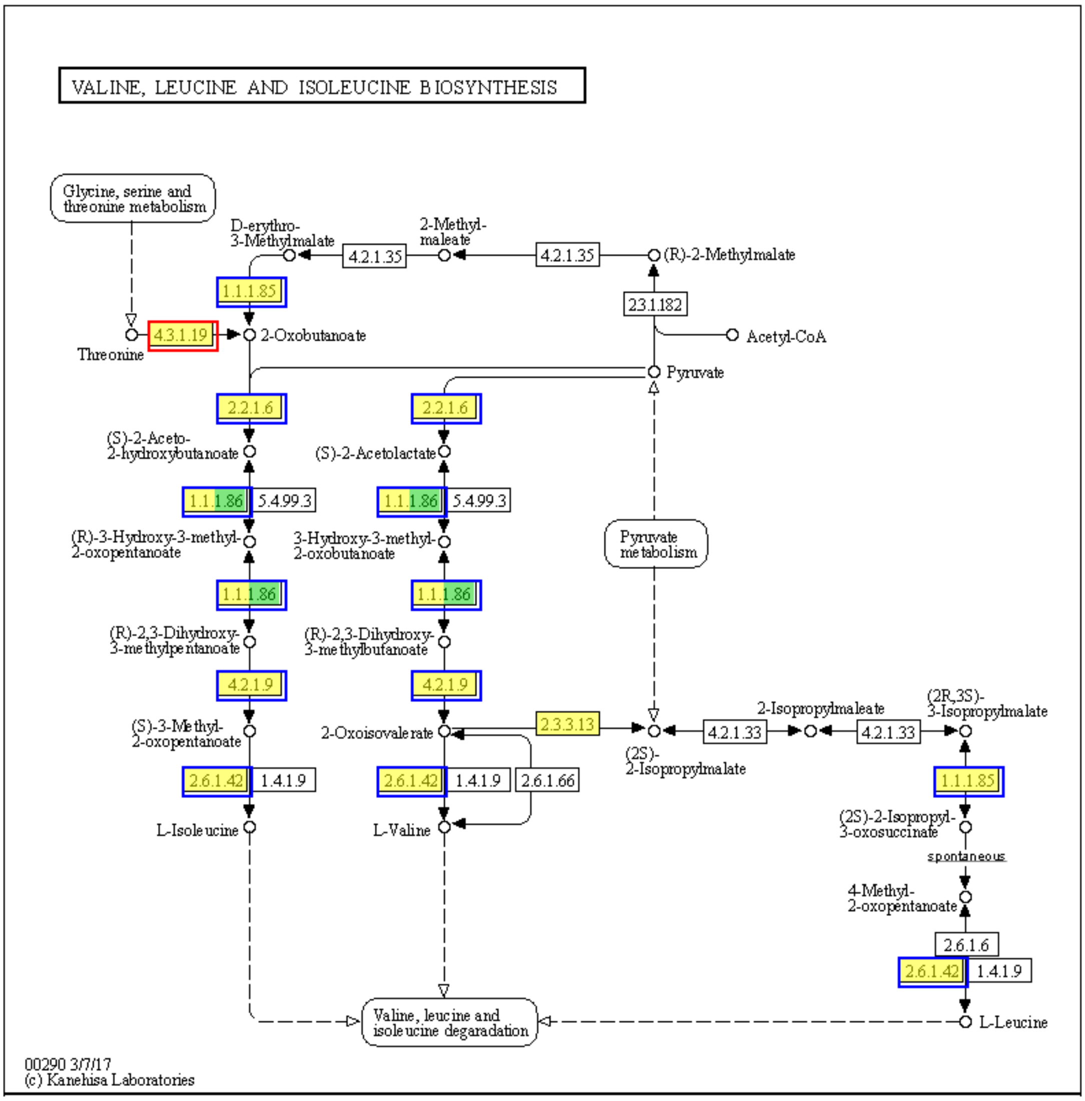

Figure S3. Valine, leucine and isoleucine biosynthesis pathway enriched by significantly different gene in control vs metalaxyl group.

Notes: Blue represents downregulated genes; Red represents upregulated genes.

Table S13. The information of DEGs in enriched valine, leucine and isoleucine biosynthesis pathway in control vs metalaxyl group.

Number	Gene ID	gene name	KO name	Pfam Description	log2FC
1	gene10025	SPRG_08786	ilvD	Dehydratase family	-1.98
2	gene2383	SPRG_01903	E2.6.1.42, ilvE	Amino-transferase class IV	-2.99
3	gene13305	SPRG_12259	E4.3.1.19, ilvA, tdcB	Pyridoxal-phosphate dependent enzyme;ACT domain	1.38
4	gene6566	SPRG_06044	ilvC	Acetohydroxy acid isomeroreductase, catalytic domain	-1.94
5	gene15673	SPRG_14440	ilvC	Acetohydroxy acid isomeroreductase, catalytic domain;Acetohydroxy acid isomeroreductase, NADPH-binding domain	-2.66
6	gene2969	SPRG_02477	leuB, IMDH	Isocitrate/isopropylmalate dehydrogenase	-1.54
7	gene5003	SPRG_04657	ilvC	Acetohydroxy acid isomeroreductase, catalytic	-1.96

				domain;Acetohydroxy acid isomeroreductase, NADPH-binding domain	
8	gene10024	SPRG_08785	ilvD	Dehydratase family	-1.20
9	gene9616	SPRG_08366	E2.2.1.6L, ilvB, ilvG, ilvI	Thiamine pyrophosphate enzyme, N-terminal TPP binding domain;Thiamine pyrophosphate enzyme, C-terminal TPP binding domain;Thiamine pyrophosphate enzyme, central domain	-1.45
10	gene6572	SPRG_05378	E2.2.1.6S, ilvH, ilvN	Small subunit of acetolactate synthase;ACT domain;ACT domain	-1.54

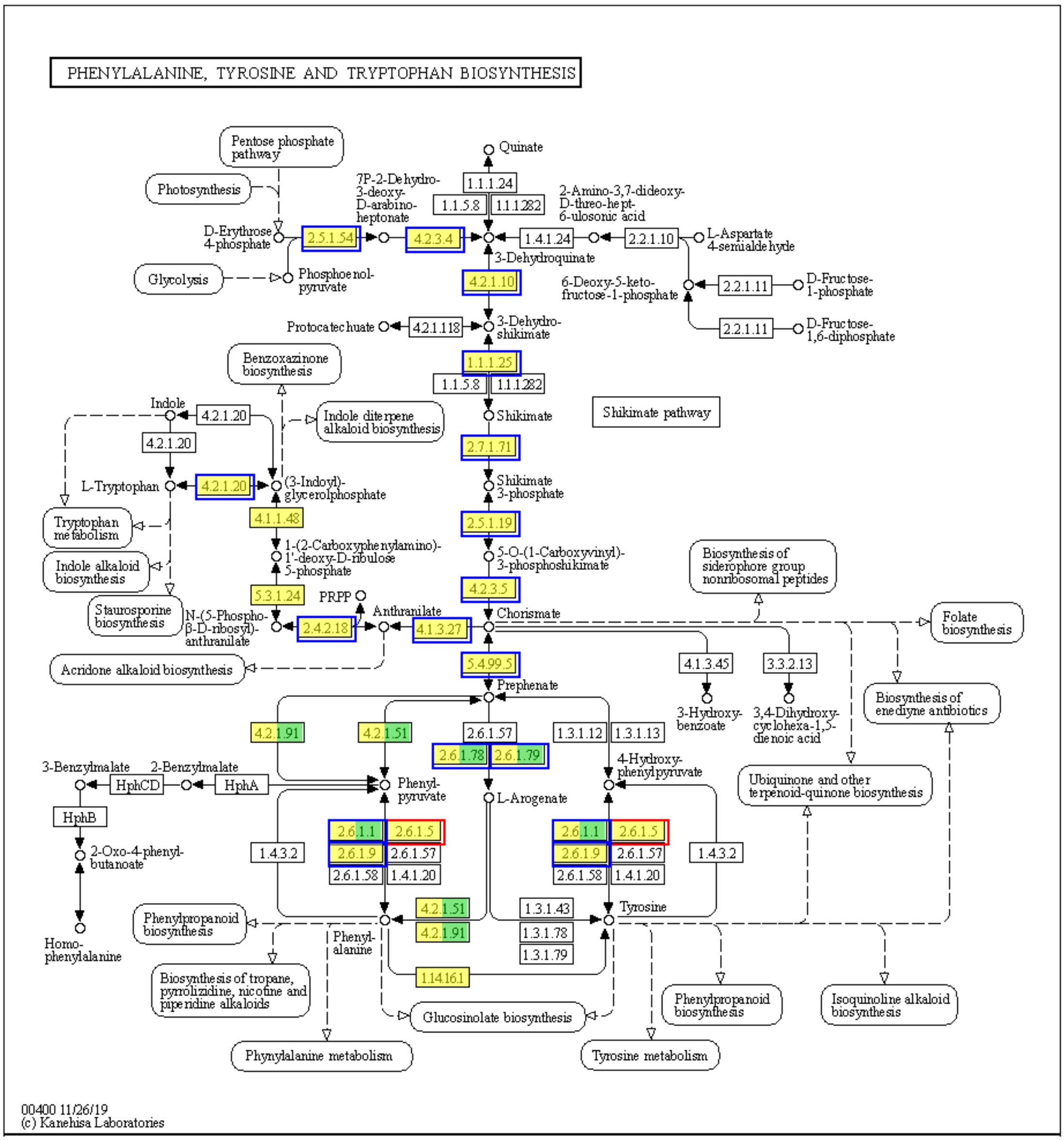

Figure S4. Phenylalanine, tyrosine and tryptophan biosynthesis pathway enriched by significantly different gene in control vs metalaxyl group.

Notes: Blue represents downregulated genes; Red represents upregulated genes.

Table S14. The information of DEGs in enriched phenylalanine, tyrosine and tryptophan biosynthesis pathway in control vs metalaxyl group.

Number	Gene ID	gene name	KO name	Pfam Description	log2FC
1	gene2017	SPRG_01617	aroC	Chorismate synthase	-1.87
2	gene19048	SPRG_17560	E2.5.1.54, aroF, aroG, aroH	DAHP synthetase I family	-1.48
3	gene9855	SPRG_08603	trpD	Glycosyl transferase family, a/b domain;Glycosyl transferase family, helical bundle domain	-1.54
4	gene7685	SPRG_20135	PAT, AAT	Aminotransferase class I and II;Prephenate dehydratase	-1.45
5	gene2736	SPRG_02249	TRP	Pyridoxal-phosphate dependent enzyme	-1.32
6	gene5831	SPRG_05285	E5.4.99.5	Prephenate dehydratase;ACT domain	-1.81
7	gene6777	SPRG_06062	trpE	chorismate binding enzyme;Anthranilate synthase component I, N terminal region	-1.65
8	gene831	SPRG_00707	E2.5.1.54, aroF, aroG, aroH	DAHP synthetase I family	-1.43
9	gene5624	SPRG_05079	TRP;	Tryptophan synthase alpha chain	-1.28
10	gene16260	SPRG_15106	hisC	Aminotransferase class I and II;Cys/Met metabolism PLP-dependent enzyme	-1.07
11	gene7858	SPRG_06771	TAT	Aminotransferase class I and II;Cys/Met metabolism PLP-dependent enzyme	3.52
12	gene9102	SPRG_08112	ARO1	EPSP synthase;3-dehydroquinate synthase;Type I 3-dehydroquinase;Shikimate kinase;Shikimate dehydrogenase substrate binding domain;Iron-containing alcohol dehydrogenase;Shikimate 5'-dehydrogenase C-terminal domain;Iron-containing alcohol dehydrogenase;Shikimate / quinate 5-dehydrogenase	-2.39
13	gene4328	hypothetical protein	E2.5.1.54, aroF, aroG, aroH	none	-1.47

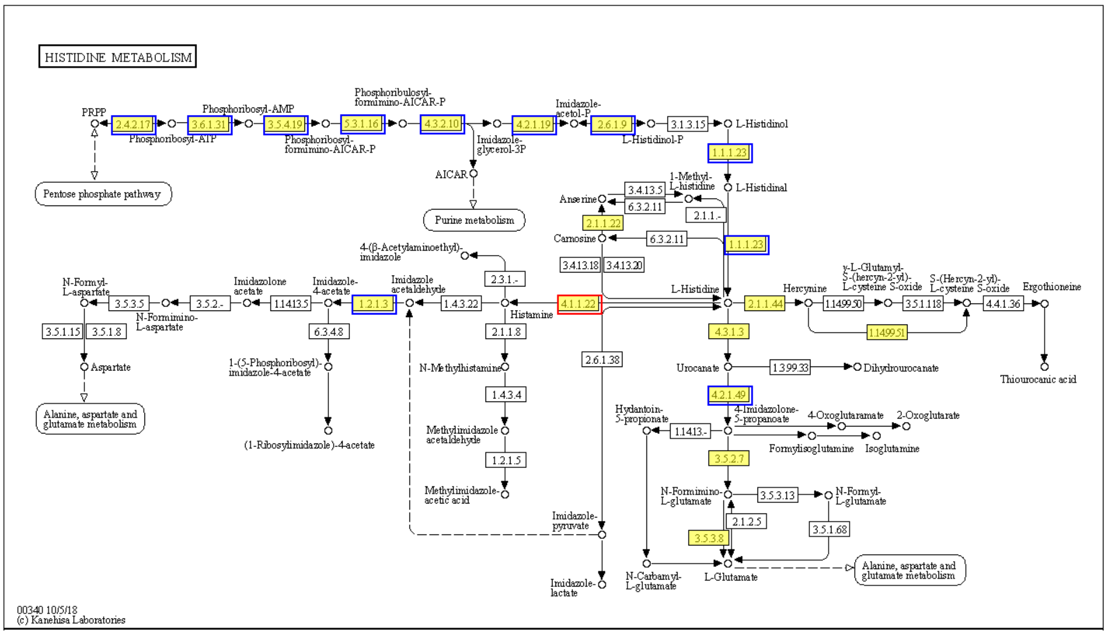

Figure S5. Histidine metabolism pathway enriched by significantly different gene in control vs metalaxyl group.

Notes: Blue represents downregulated genes; Red represents upregulated genes.

Table S15. The information of DEGs in enriched histidine metabolism pathway in control vs metalaxyl group.

Number	Gene ID	gene name	KO name	Pfam Description	log2FC
1	gene7118	SPRG_05764	hisG	ATP phosphoribosyltransferase;HisG, C-terminal domain	-2.08
2	gene19525	SPRG_17954	ALDH	Aldehyde dehydrogenase family	-3.28
3	gene2354	SPRG_01876	HIS4	Histidinol dehydrogenase	-2.06
4	gene455	SPRG_00441	HIS7	Histidine biosynthesis protein;Glutamine amidotransferase class-I;SNO glutamine amidotransferase family;CobB/CobQ-like glutamine amidotransferase domain;Peptidase C26	-1.97
5	gene15249	SPRG_13327	HIS4	Phosphoribosyl-AMP cyclohydrolase;Phosphoribosyl-ATP pyrophosphohydrolase	-1.95
6	gene814	SPRG_00691	hisB	Imidazoleglycerol-phosphate dehydratase;Haloacid dehalogenase-like hydrolase;haloacid dehalogenase-like hydrolase	-1.83
7	gene5371	SPRG_04491	hutU, UROC1	Urocanase C-terminal domain;Urocanase Rossmann-like domain;Urocanase N-terminal domain	-1.25
8	gene16260	SPRG_15106	hisC	Aminotransferase class I and II;Cys/Met metabolism PLP-dependent enzyme	-1.07
9	gene9708	SPRG_08456	ALDH	Aldehyde dehydrogenase family	-1.20
10	gene9707	SPRG_08455	ALDH	Aldehyde dehydrogenase family	-2.18
11	gene15559	SPRG_14345	hisA	Histidine biosynthesis protein	-1.07
12	gene14180	SPRG_12642	hdc, HDC	Pyridoxal-dependent decarboxylase conserved domain;L-seryl-tRNA selenium transferase	6.62

13	gene18172	SPRG_16717	hisG	ATP phosphoribosyltransferase;HisG, C-terminal domain	-1.73

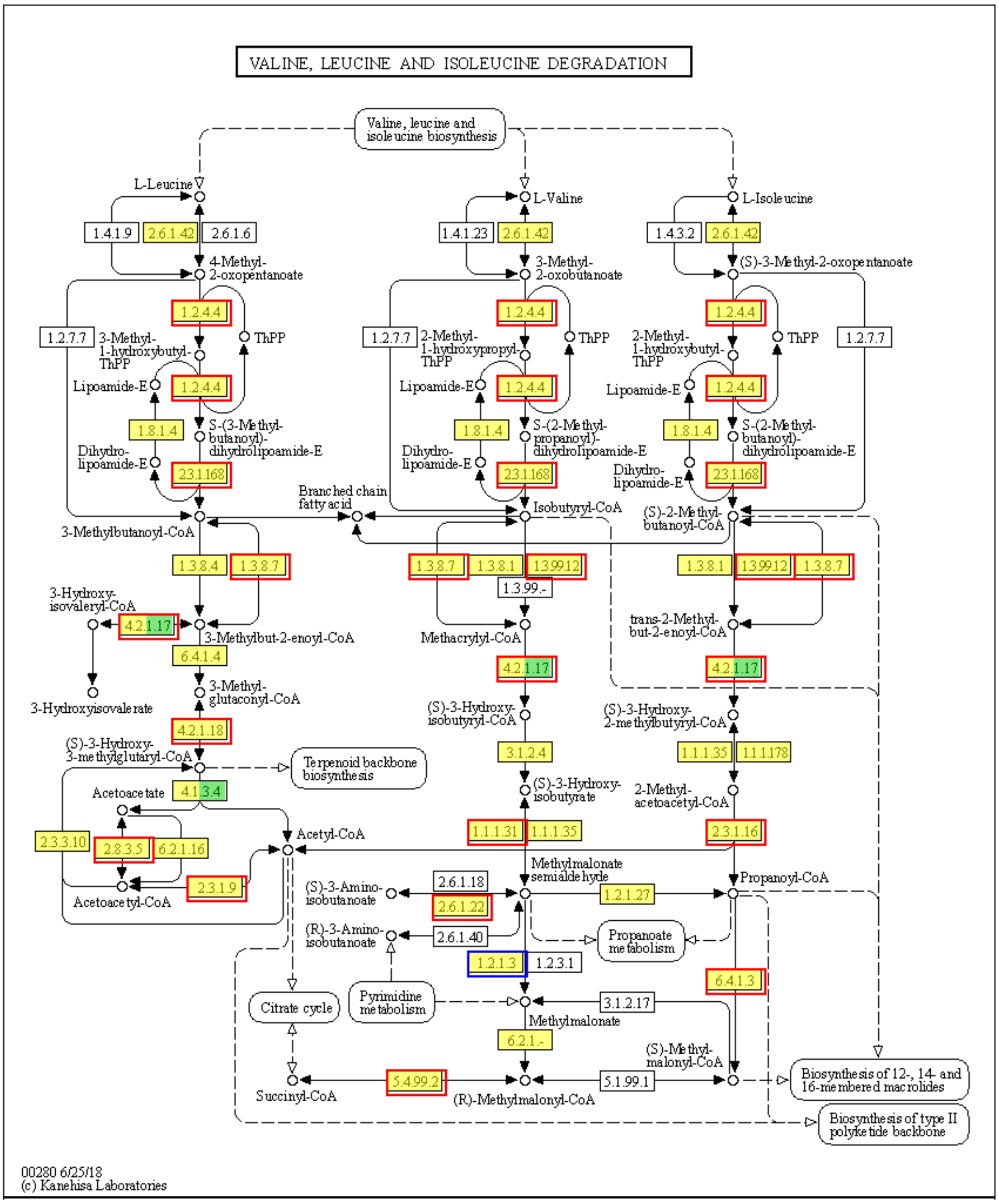

Figure S6. Valine, leucine and isoleucine degradation pathway enriched by significantly different gene in control vs bronopol group.

Notes: Blue represents downregulated genes; Red represents upregulated genes.

Table S16. The information of DEGs in enriched valine, leucine and isoleucine degradation pathway in control vs bronopol group.

Number	Gene ID	gene name	KO name	Pfam Description	log2FC
1	gene56	SPRG_00057	PCCA, pccA	Carbamoyl-phosphate synthase L chain, ATP binding domain; Biotin carboxylase, N-terminal domain; Biotin carboxylase C-terminal domain; Biotin-requiring enzyme; Propionyl-coenzyme A carboxylase BT domain; ATP-grasp domain; D-ala D-ala ligase C-terminus	1.10
2	gene8186	SPRG_07368	HADHB	Thiolase, N-terminal domain; Thiolase, C-terminal domain	1.74
3	gene4718	SPRG_04185	DBT, bkdB	2-oxoacid dehydrogenases acyltransferase; Biotin-requiring enzyme; e3 binding domain; Biotin-lipoyl like	1.53
4	gene4719	SPRG_04186	BCKDHB, bkdA2	Transketolase, pyrimidine binding domain; Transketolase, C-terminal domain	1.54
5	gene12557	SPRG_11495	ABAT	Aminotransferase class-III	1.76
6	gene4196	SPRG_03679	ACADSB	Acyl-CoA dehydrogenase, C-terminal domain; Acyl-CoA dehydrogenase, N-terminal domain; Acyl-CoA dehydrogenase, middle domain; Acyl-CoA dehydrogenase, C-terminal domain	2.06
7	gene1203	SPRG_01080	AUH	Enoyl-CoA hydratase/isomerase; Enoyl-CoA hydratase/isomerase	2.26
8	gene17358	SPRG_16016	MUT	Methylmalonyl-CoA mutase; B12 binding domain	1.06
9	gene14775	SPRG_11033	MUT	Methylmalonyl-CoA mutase; B12 binding domain	1.05
10	gene9708	SPRG_08456	ALDH	Aldehyde dehydrogenase family	-1.46
11	gene9707	SPRG_08455	ALDH	Aldehyde dehydrogenase family	-2.02
12	gene10214	SPRG_09078	HIBADH, mmsB	NAD binding domain of 6-phosphogluconate dehydrogenase; NAD-binding of NADP-dependent 3-hydroxyisobutyrate dehydrogenase; D-isomer specific 2-hydroxyacid dehydrogenase, NAD binding domain; NADP oxidoreductase coenzyme F420-dependent;3-hydroxyacyl-CoA dehydrogenase, NAD binding domain; TrkA-N domain	1.35
13	gene260	SPRG_00258	E2.3.1.9, atoB	Thiolase, N-terminal domain; Thiolase, C-terminal domain; Beta-ketoacyl synthase, N-terminal domain	1.28
14	gene19608	SPRG_18022	ACAA2	Thiolase, N-terminal domain	1.91
15	gene9565	SPRG_08315	paaF, echA	Enoyl-CoA hydratase/isomerase; Enoyl-CoA hydratase/isomerase	1.57
16	gene8448	SPRG_06986	ACADM, acd	Acyl-CoA dehydrogenase, C-terminal domain; Acyl-CoA dehydrogenase, middle domain; Acyl-CoA dehydrogenase, N-terminal domain; Acyl-CoA dehydrogenase, C-terminal domain; 4-hydroxyphenylacetate 3-hydroxylase N terminal	1.32
17	gene12395	SPRG_10775	OXCT	Coenzyme A transferase	1.38
18	gene16413	SPRG_15243	ACAA2	Thiolase, N-terminal domain; Thiolase, C-terminal domain	1.58
19	gene7358	SPRG_06557	HADHA	3-hydroxyacyl-CoA dehydrogenase, NAD binding domain; Enoyl-CoA hydratase/isomerase; 3-hydroxyacyl-CoA dehydrogenase, C-terminal domain; Enoyl-CoA hydratase/isomerase	1.32

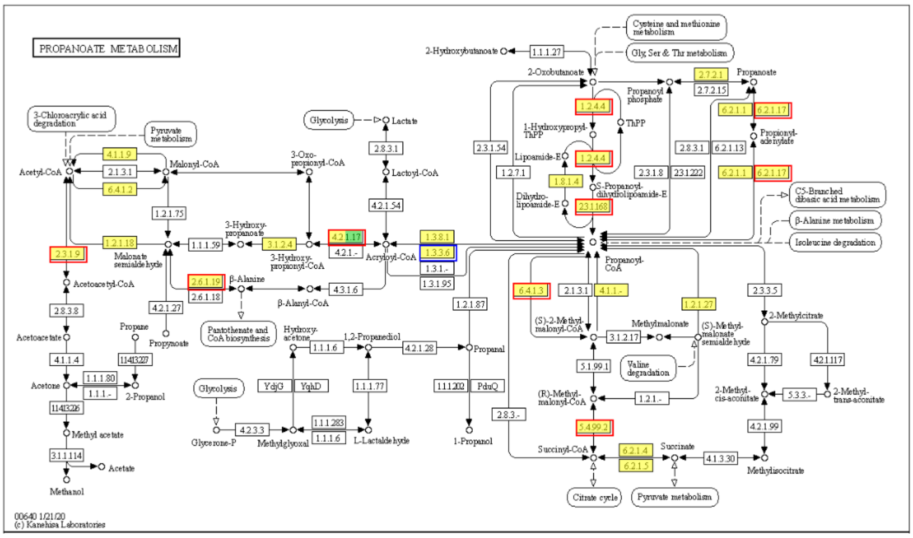

Figure S7. Propanoate metabolism pathway enriched by significantly different gene in control vs bronopol group.

Notes: Blue represents downregulated genes; Red represents upregulated genes.

Table S17. The information of DEGs in enriched propanoate metabolism pathway in control vs bronopol group.

Number	Gene ID	gene name	KO name	Pfam Description	log2FC
1	gene56	SPRG_00057	PCCA, pccA	Carbamoyl-phosphate synthase L chain, ATP binding domain; Biotin carboxylase, N-terminal domain;Biotin carboxylase C-terminal domain;Biotin-requiring enzyme;Propionyl-coenzyme A carboxylase BT domain;ATP-grasp domain; D-ala D-ala ligase C-terminus	1.13
2	gene4718	SPRG_04185	DBT, bkdB	2-oxoacid dehydrogenases acyltransferase;Biotin-requiring enzyme;e3 binding domain;Biotin-lipoyl like	1.50
3	gene4719	SPRG_04186	BCKDHB , bkdA2	Transketolase, pyrimidine binding domain;Transketolase, C-terminal domain	1.47
4	gene12557	SPRG_11495	ABAT	Aminotransferase class-III	-0.93
5	gene13165	SPRG_11585	E1.3.3.6, ACOX1, ACOX3	Acyl-CoA oxidase; Acyl-coenzyme A oxidase N-terminal; Acyl-CoA dehydrogenase, middle domain	-0.91
6	gene17358	SPRG_16016	MUT	Methylmalonyl-CoA mutase; B12 binding domain	0.77
7	gene14775	SPRG_11033	MUT	Methylmalonyl-CoA mutase; B12 binding domain	0.67
8	gene260	SPRG_00258	E2.3.1.9, atoB	Thiolase, N-terminal domain;Thiolase, C-terminal domain;Beta-ketoacyl synthase, N-terminal domain	1.16
9	gene9039	SPRG_08067	ACSS3, prpE	AMP-binding enzyme; Acetyl-coenzyme A synthetase N-terminus; AMP-binding enzyme C-terminal domain	0.29
10	gene9565	SPRG_08315	paaF, echA	Enoyl-CoA hydratase/isomerase; Enoyl-CoA hydratase/isomerase	1.12
11	gene7358	SPRG_06557	HADHA	3-hydroxyacyl-CoA dehydrogenase, NAD binding domain; Enoyl-CoA hydratase/isomerase;3-hydroxyacyl-CoA dehydrogenase, C-terminal	1.09

				domain; Enoyl-CoA hydratase/isomerase	

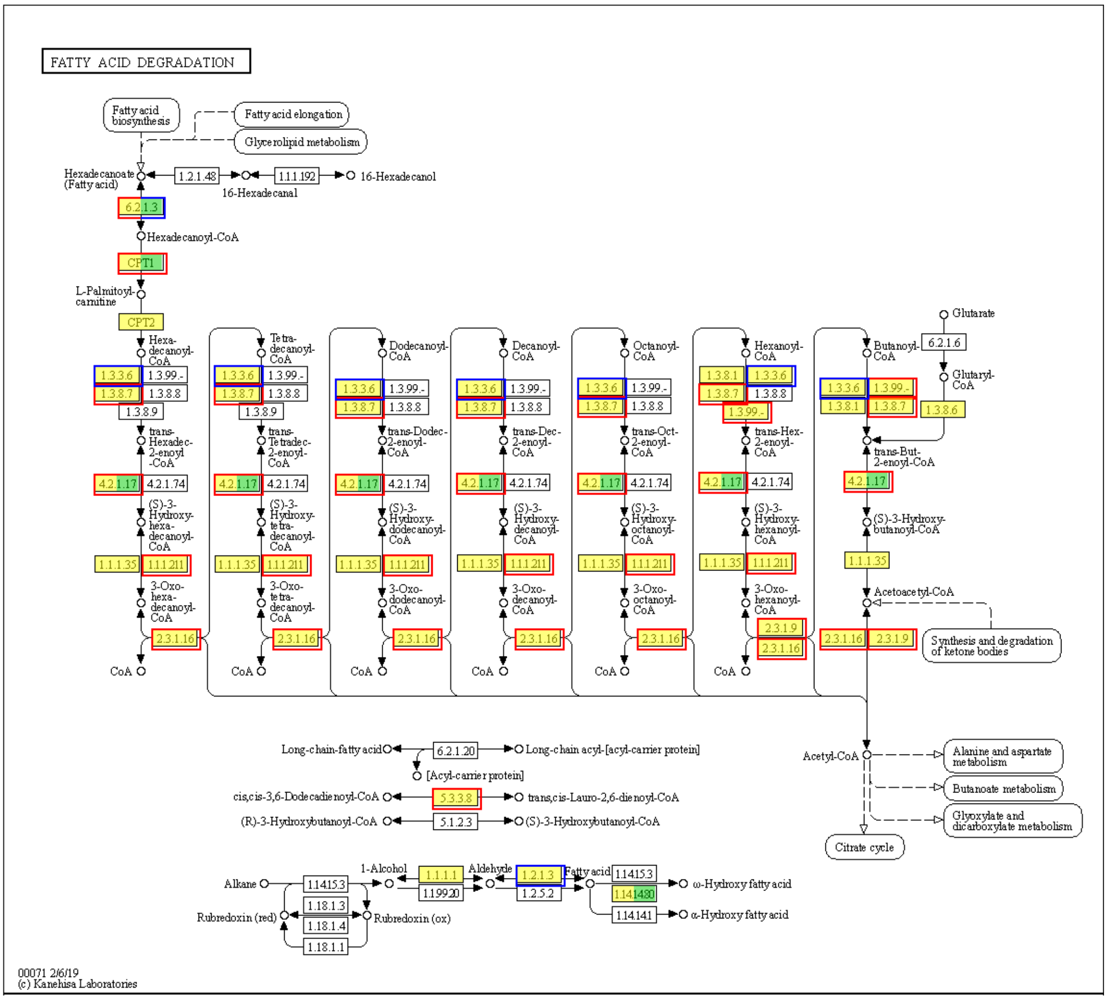

Figure S8. Fatty acid degradation pathway enriched by significantly different gene in control vs bronopol group.

Notes: Blue represents downregulated genes; Red represents upregulated genes.

Table S18. The information of DEGs in enriched fatty acid degradation pathway in control vs bronopol group.

Number	Gene ID	gene name	KO name	Pfam Description	log2FC
1	gene17997	SPRG_16549	ACSL, fadD	AMP-binding enzyme	-1.37
2	gene8186	SPRG_07368	HADHB	Thiolase, N-terminal domain; Thiolase, C-terminal domain	1.74
3	gene4196	SPRG_03679	ACADSB	Acyl-CoA dehydrogenase, C-terminal domain; Acyl-CoA dehydrogenase, N-terminal domain; Acyl-CoA dehydrogenase, middle domain; Acyl-CoA dehydrogenase, C-terminal domain	2.06
4	gene13165	SPRG_11585	E1.3.3.6, ACOX1, ACOX3	Acyl-CoA oxidase; Acyl-coenzyme A oxidase N-terminal; Acyl-CoA dehydrogenase, middle domain	-1.08
5	gene3685	SPRG_03174	ECI1, DCI	Enoyl-CoA hydratase/isomerase; Enoyl-CoA hydratase/isomerase	1.07
6	gene4675	SPRG_04143	ACSL, fadD	AMP-binding enzyme	4.15

7	gene9708	SPRG_08456	ALDH	Aldehyde dehydrogenase family	-1.46
8	gene9707	SPRG_08455	ALDH	Aldehyde dehydrogenase family	-2.02
9	gene260	SPRG_00258	E2.3.1.9, atoB	Thiolase, N-terminal domain; Thiolase, C-terminal domain; Beta-ketoacyl synthase, N-terminal domain	1.28
10	gene2744	SPRG_02257	CPT1A	Choline/Carnitine o-acyltransferase	2.94
11	gene19608	SPRG_18022	ACAA2	Thiolase, N-terminal domain	1.91
12	gene12003	SPRG_10022	ECI2, PECI	Enoyl-CoA hydratase/isomerase; Acyl CoA binding protein; Enoyl-CoA hydratase/isomerase	1.17
13	gene9565	SPRG_08315	paaF, echA	Enoyl-CoA hydratase/isomerase; Enoyl-CoA hydratase/isomerase	1.57
14	gene8448	SPRG_06986	ACADM, acd	Acyl-CoA dehydrogenase, C-terminal domain; Acyl-CoA dehydrogenase, middle domain; Acyl-CoA dehydrogenase, N-terminal domain; Acyl-CoA dehydrogenase, C-terminal domain;4-hydroxyphenylacetate 3-hydroxylase N terminal	1.32
15	gene16413	SPRG_15243	ACAA2	Thiolase, N-terminal domain; Thiolase, C-terminal domain	1.58
16	gene7358	SPRG_06557	HADHA	3-hydroxyacyl-CoA dehydrogenase, NAD binding domain; Enoyl-CoA hydratase/isomerase;3-hydroxyacyl-CoA dehydrogenase, C-terminal domain; Enoyl-CoA hydratase/isomerase	1.32

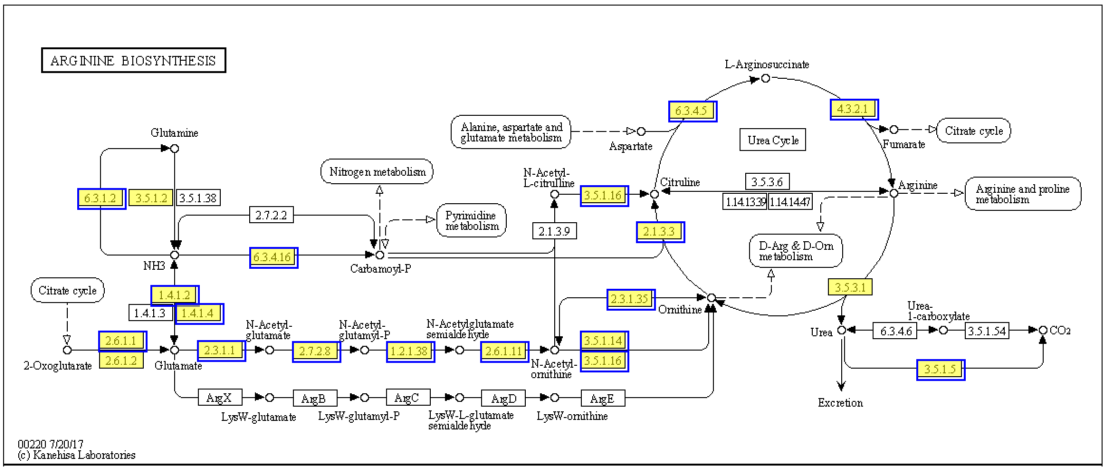

Figure S9. Arginine biosynthesis pathway enriched by significantly different gene in control vs copper sulfate.

Notes: Blue represents downregulated genes; Red represents upregulated genes.

Table S19. The information of DEGs in enriched arginine biosynthesis pathway in control vs copper sulfate.

Number	Gene ID	gene name	KO name	Pfam Description	log2FC
1	gene8710	SPRG_07573	argAB	Amino acid kinase family;Acetyltransferase;Acetyltransferase;Acetyltransferase	-1.87
2	gene4540	SPRG_04013	argE	Peptidase family M20/M25/M40;Peptidase dimerisation domain	-1.15
3	gene1560	SPRG_01415	ACY1	Peptidase family M20/M25/M40;Peptidase dimerisation	-1.60

				domain;Peptidase family M28	
4	gene781	SPRG_00658	E2.6.1.11, argD	Aminotransferase class-III	-1.20
5	gene7478	SPRG_06677	argJ	ArgJ family	-1.93
6	gene3384	SPRG_02883	glnA, GLUL	Glutamine synthetase, catalytic domain;Glutamine synthetase, beta-Grasp domain	-1.15
7	gene6663	SPRG_05438	CPS1	Carbamoyl-phosphate synthase L chain, ATP binding domain;Glutamine amidotransferase class-I;Carbamoyl-phosphate synthase small chain, CPSase domain;Carbamoyl-phosphate synthetase large chain, oligomerisation domain;D-ala D-ala ligase C-terminus;ATP-grasp domain	-1.74
8	gene6665	SPRG_05439	ARG56;	Amino acid kinase family;Semialdehyde dehydrogenase, NAD binding domain;Semialdehyde dehydrogenase, dimerisation domain	-1.83
9	gene7825	SPRG_06768	glnA, GLUL	Glutamine synthetase, catalytic domain;Glutamine synthetase, beta-Grasp domain	-1.53
10	gene7824	SPRG_20244	glnA, GLUL	Glutamine synthetase, catalytic domain	-1.75
11	gene2133	SPRG_01659	E1.4.1.4, gdhA	Glutamate/Leucine/Phenylalanine/Valine dehydrogenase;Glu/Leu/Phe/Val dehydrogenase, dimerisation domain	-1.30
12	gene834	SPRG_00710	GOT2	Aminotransferase class I and II	-1.17
13	gene3092	SPRG_02592	argG, ASS1	Arginosuccinate synthase	-1.58
14	gene6905	SPRG_06160	argH, ASL	Lyase;Argininosuccinate lyase C-terminal	-2.45
15	gene8267	SPRG_06801	URE	Amidohydrolase family;Urease alpha-subunit, N-terminal domain;Urease, gamma subunit;Urease beta subunit;Amidohydrolase family	-1.89
16	gene3094	SPRG_19437	argG, ASS1	Arginosuccinate synthase	-1.43
17	gene19263	SPRG_17724	ARG56;K22478	Semialdehyde dehydrogenase, NAD binding domain	-2.30
18	gene4383	SPRG_03863	OTC, argF, argI	Aspartate/ornithine carbamoyltransferase, carbamoyl-P binding domain;Aspartate/ornithine carbamoyltransferase, Asp/Orn binding domain	-2.80
19	gene20243	SPRG_18639	GDH2	Glutamate/Leucine/Phenylalanine/Valine dehydrogenase;Bacterial NAD-glutamate dehydrogenase	-1.33
20	gene3183	SPRG_02683	GDH2	Glutamate/Leucine/Phenylalanine/Valine dehydrogenase;Bacterial NAD-glutamate dehydrogenase	-1.65

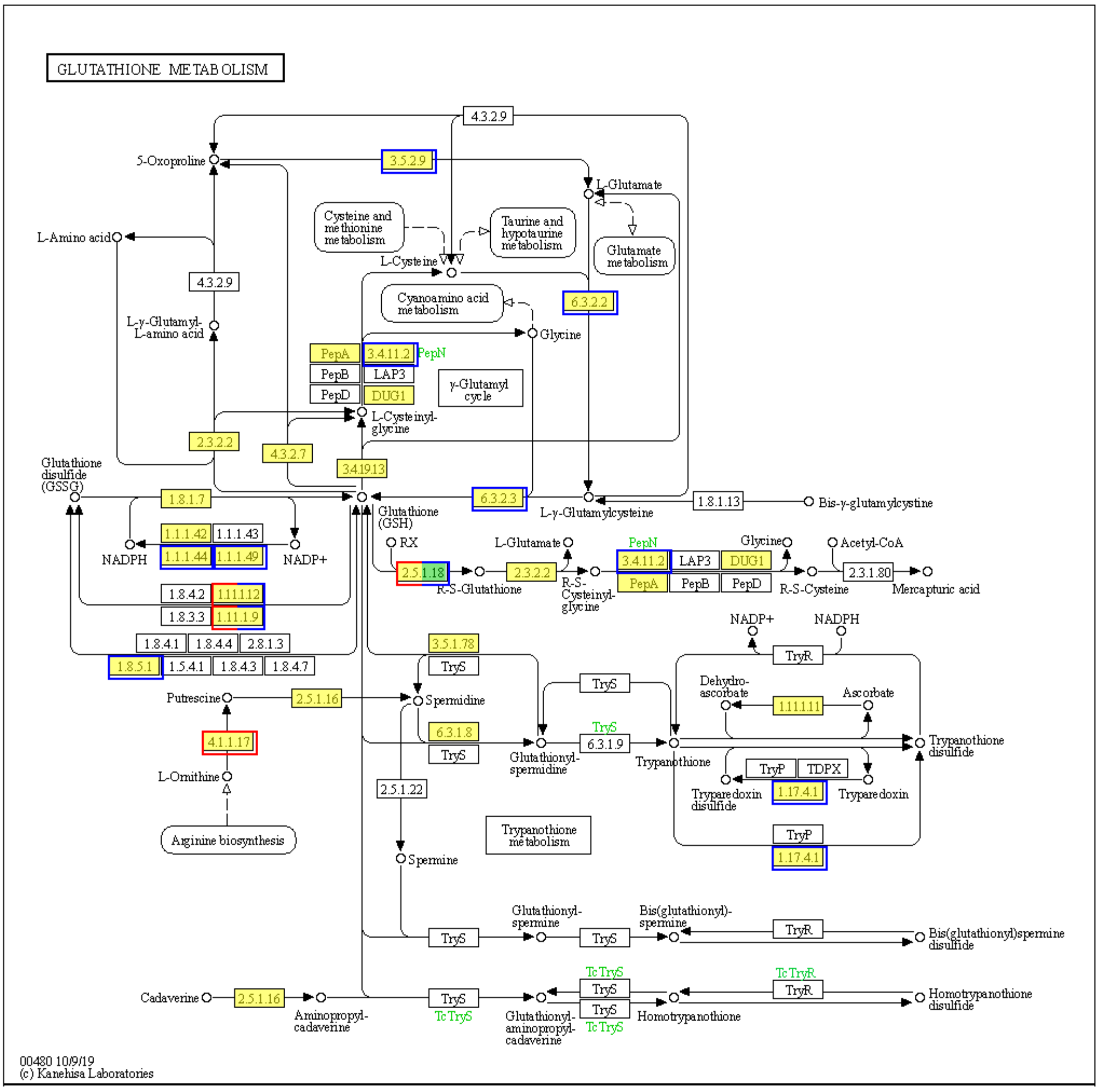

Figure S10. Glutathione metabolism pathway enriched by significantly different gene in control vs copper sulfate.

Notes: Blue represents downregulated genes; Red represents upregulated genes.

Table S20. The information of DEGs in enriched glutathione metabolism pathway in control vs copper sulfate.

Number	Gene ID	gene name	KO name	Pfam Description	log2FC
1	gene5812	SPRG_05266	G6PD, zwf	Glucose-6-phosphate dehydrogenase, C-terminal domain; Glucose-6-phosphate dehydrogenase, NAD binding domain	-1.02
2	gene14097	SPRG_12363	DHAR	Glutathione S-transferase, C-terminal domain; Glutathione S-transferase, N-terminal domain;Glutathione S-transferase, C-terminal domain;Glutathione S-transferase, N-terminal domain;Glutathione S-transferase, N-terminal domain;Glutathione S-transferase, C-terminal domain	-1.32

3	gene14098	SPRG_12364	DHAR	Glutathione S-transferase, C-terminal domain;Glutathione S-transferase, N-terminal domain;Glutathione S-transferase, N-terminal domain;Glutathione S-transferase, C-terminal domain;Glutathione S-transferase, N-terminal domain;Glutathione S-transferase, C-terminal domain	-3.41
4	gene514	SPRG_18820	;GST, gst	MAPEG family	-4.37
5	gene10676	SPRG_09447	GSS	Eukaryotic glutathione synthase, ATP binding domain;Eukaryotic glutathione synthase	-1.09
6	gene10469	SPRG_09358	HPGDS	Glutathione S-transferase, C-terminal domain;Glutathione S-transferase, N-terminal domain;Glutathione S-transferase, C-terminal domain	-1.63
7	gene7600	SPRG_06230	HPGDS	Glutathione S-transferase, C-terminal domain	7.21
8	gene6929	SPRG_06183	GST, gst	MAPEG family	-1.25
9	gene6928	SPRG_06182	GST, gst	MAPEG family	2.44
10	gene14539	SPRG_12421	G6PD, zwf	Glucose-6-phosphate dehydrogenase, C-terminal domain;Glucose-6-phosphate dehydrogenase, NAD binding domain	-1.99
11	gene9814	SPRG_08563	PGD, gnd, gntZ	6-phosphogluconate dehydrogenase, C-terminal domain;NAD binding domain of 6-phosphogluconate dehydrogenase	-1.00
12	gene2275	SPRG_01800	GPX4;gpx, btuE, bsaA	Glutathione peroxidase	-1.16
13	gene7865	SPRG_20277	HPGDS	Glutathione S-transferase, C-terminal domain;Glutathione S-transferase, N-terminal domain;Glutathione S-transferase, N-terminal domain;Glutathione S-transferase, C-terminal domain	-1.16
14	gene17946	SPRG_16543	HPGDS	Glutathione S-transferase, C-terminal domain;Glutathione S-transferase, C-terminal domain;Glutathione S-transferase, N-terminal domain;Glutathione S-transferase, C-terminal domain	1.53
15	gene4084	SPRG_03567	GCLC	Glutamate-cysteine ligase	-3.11
16	gene15352	SPRG_12960	HPGDS	Glutathione S-transferase, C-terminal domain;Glutathione S-transferase, C-terminal domain;Glutathione S-transferase, N-terminal domain	1.84
17	gene4646	SPRG_04116	RRM1	Ribonucleotide reductase, barrel domain;Ribonucleotide reductase, all-alpha domain;ATP cone domain	-2.14
18	gene9670	SPRG_08418	GST, gst	MAPEG family	-2.21
19	gene16910	SPRG_15022	HPGDS;PIR	Glutathione S-transferase, C-terminal domain	-1.37
20	gene13584	SPRG_11344	HPGDS;PIR	Glutathione S-transferase, C-terminal domain;Glutathione S-transferase, N-terminal domain;Glutathione S-transferase, C-terminal domain	5.85
21	gene18969	SPRG_17314	GST, gst	MAPEG family	-1.92
22	gene2633	SPRG_02146	OPLAH, OXP1, oplAH	Hydantoinase B/oxoprolinase; Hydantoinase/oxoprolinase; Hydantoinase/oxoprolinase N-terminal region	-1.30
23	gene7599	SPRG_22185	HPGDS;PIR	Glutathione S-transferase, C-terminal domain; Glutathione S-transferase, C-terminal domain; Glutathione S-transferase, C-terminal domain	5.88
24	gene4590	SPRG_04061	pepN	Peptidase family M1 domain; Domain of unknown function;	-3.15

				Peptidase M1 N-terminal domain; Domain of unknown function	
25	gene7866	SPRG_20278	HPGDS	Glutathione S-transferase, C-terminal domain; Glutathione S-transferase, N-terminal domain	1.01
26	gene9072	SPRG_08082	gpx, btuE, bsaA;GPX4	Glutathione peroxidase	1.82
27	gene15668	SPRG_14435	RRM2	Ribonucleotide reductase, small chain	-2.70
28	gene13585	SPRG_11345	HPGDS;PIR	Glutathione S-transferase, C-terminal domain;Glutathione S-transferase, N-terminal domain	6.34
29	gene17654	SPRG_16152	DHAR	Glutathione S-transferase, C-terminal domain;Glutathione S-transferase, N-terminal domain;Glutathione S-transferase, N-terminal domain;Glutathione S-transferase, C-terminal domain;Glutathione S-transferase, N-terminal domain;Glutathione S-transferase, C-terminal domain	-3.64
30	gene19865	SPRG_18265	RRM2	Ribonucleotide reductase, small chain	-2.61
31	gene11247	SPRG_10256	GST, gst	Glutathione S-transferase, N-terminal domain;Glutathione S-transferase, N-terminal domain;Glutathione S-transferase, N-terminal domain;Glutathione S-transferase, C-terminal domain;Glutathione S-transferase, C-terminal domain	-1.71
32	gene5125	SPRG_04780	E4.1.1.17, ODC1, speC, speF	Pyridoxal-dependent decarboxylase, pyridoxal binding domain; Pyridoxal-dependent decarboxylase, C-terminal sheet domain	1.44
33	gene17653	SPRG_16151	DHAR	Glutathione S-transferase, C-terminal domain;Glutathione S-transferase, N-terminal domain; Glutathione S-transferase, C-terminal domain; Glutathione S-transferase, N-terminal domain; Glutathione S-transferase, N-terminal domain; Glutathione S-transferase, C-terminal domain	-3.31

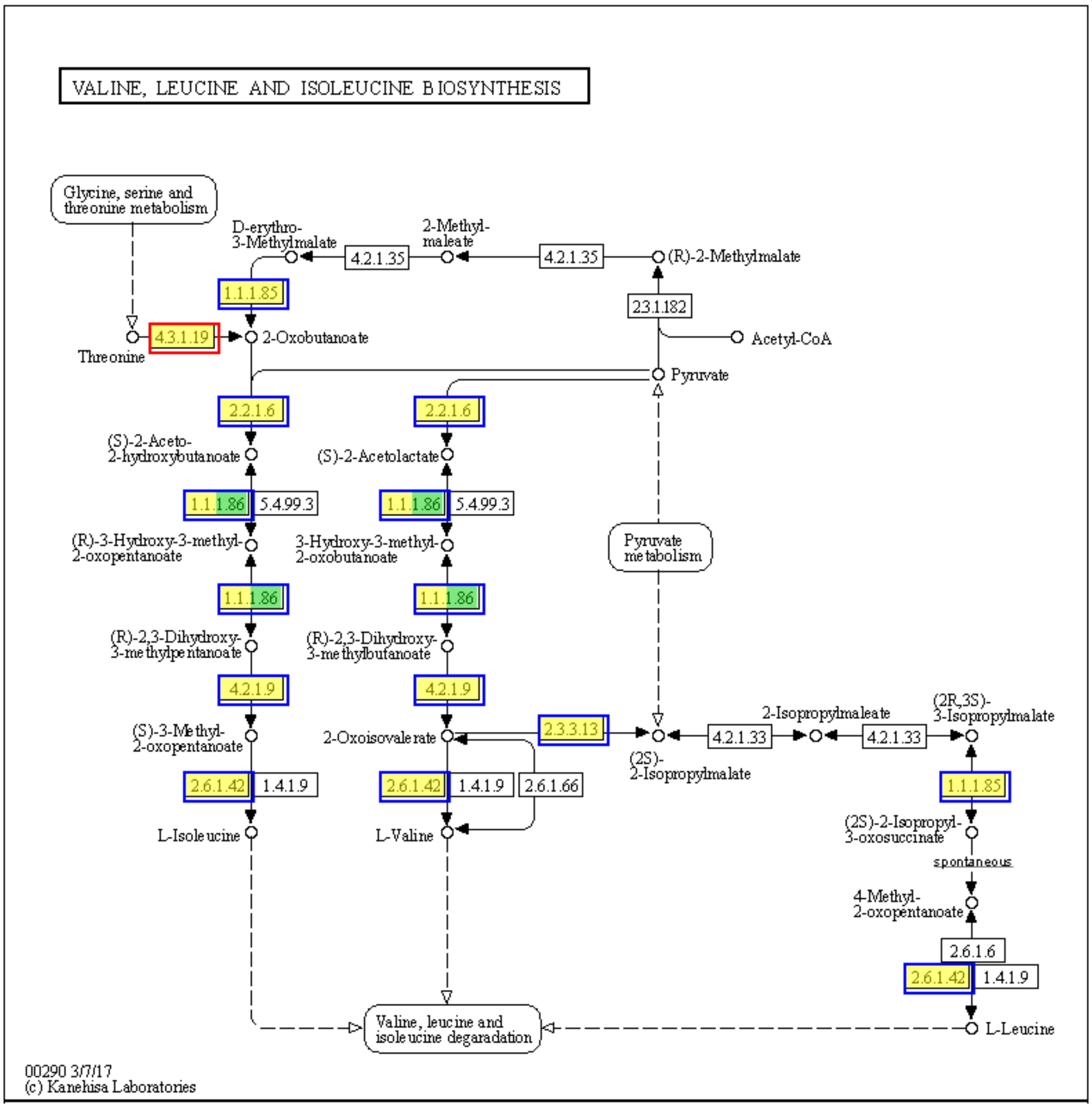

Figure S11. Valine, leucine and isoleucine biosynthesis pathway enriched by significantly different gene in control vs copper sulfate.

Notes: Blue represents downregulated genes; Red represents upregulated genes.

Table S21. The information of DEGs in enriched valine, leucine and isoleucine biosynthesis pathway in control vs copper sulfate.

Number	Gene ID	gene name	KO name	Pfam Description	log2FC
1	gene10025	SPRG_08786	ilvD	Dehydratase family	-3.15
2	gene2383	SPRG_01903	E2.6.1.42, ilvE	Amino-transferase class IV	-3.65
3	gene13305	SPRG_12259	E4.3.1.19, ilvA, tdcB	Pyridoxal-phosphate dependent enzyme; ACT domain	1.70
4	gene6566	SPRG_06044	ilvC	Acetohydroxy acid isomeroreductase, catalytic domain	-2.95
5	gene15673	SPRG_14440	ilvC	Acetohydroxy acid isomeroreductase, catalytic domain; Acetohydroxy acid isomeroreductase, NADPH-binding domain	-3.64
6	gene2969	SPRG_02477	;;leuB, IMDH	Isocitrate/isopropylmalate dehydrogenase	-2.36

7	gene5003	SPRG_04657	ilvC	Acetohydroxy acid isomeroreductase, catalytic domain; Acetohydroxy acid isomeroreductase, NADPH-binding domain	-2.66
8	gene14100	SPRG_12368	leuA, IMS	HMGL-like; LeuA allosteric	-1.15
9	gene10024	SPRG_08785	ilvD	Dehydratase family	-1.47
10	gene9616	SPRG_08366	E2.2.1.6L, ilvB, ilvG, ilvI	Thiamine pyrophosphate enzyme, N-terminal TPP binding domain; Thiamine pyrophosphate enzyme, C-terminal TPP binding domain; Thiamine pyrophosphate enzyme, central domain	-2.52
11	gene6572	SPRG_05378	E2.2.1.6S, ilvH, ilvN	Small subunit of acetolactate synthase; ACT domain;ACT domain	-1.82

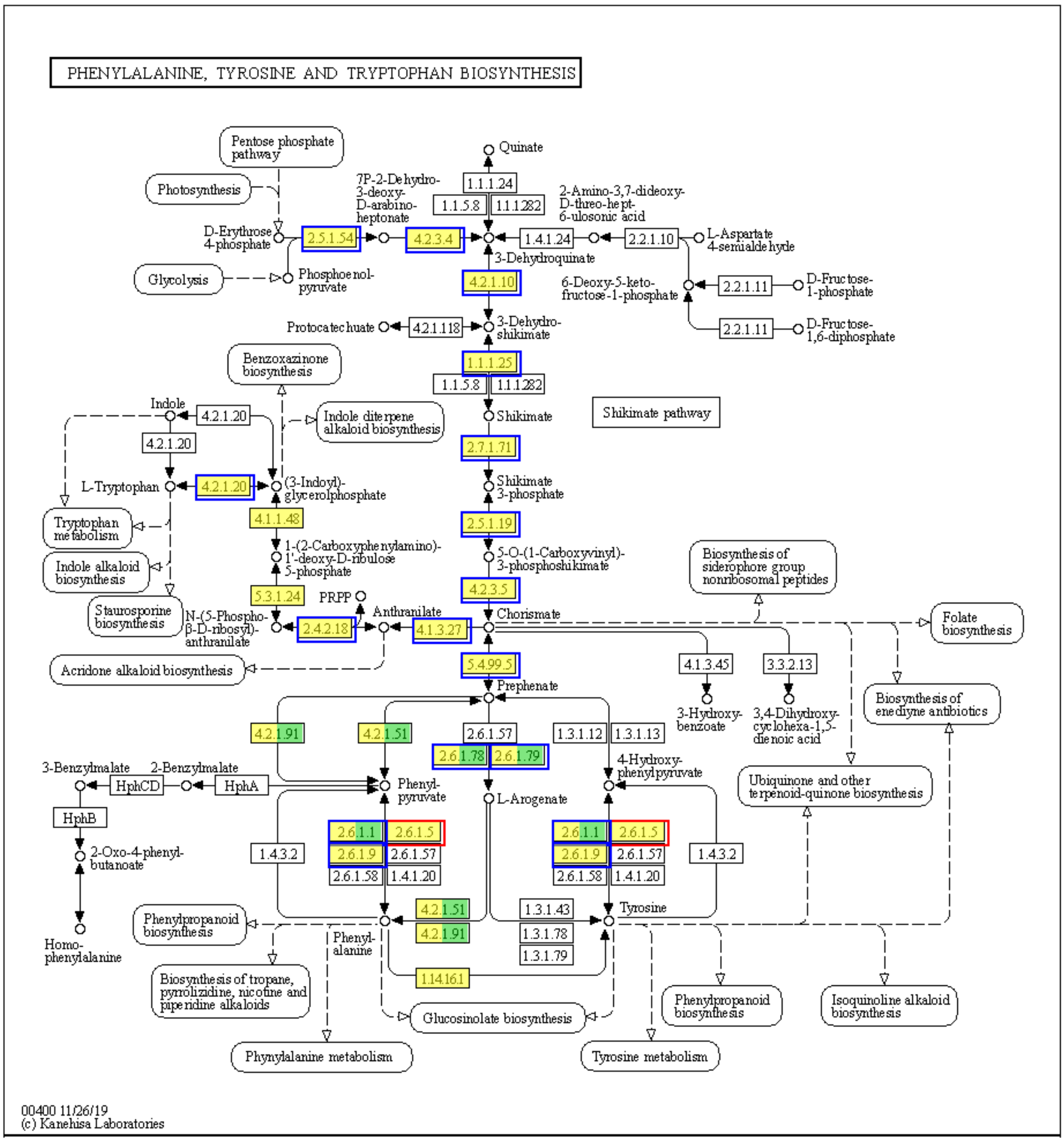

Figure S12. Phenylalanine, tyrosine and tryptophan biosynthesis pathway enriched by

significantly different gene in control vs copper sulfate.

Notes: Blue represents downregulated genes; Red represents upregulated genes.

Table S22. The information of DEGs in enriched phenylalanine, tyrosine and tryptophan biosynthesis pathway in control vs copper sulfate.

Number	Gene ID	gene name	KO name	Pfam Description	log2FC
1	gene2017	SPRG_01617	aroC	Chorismate synthase	-2.53
2	gene19048	SPRG_17560	E2.5.1.54, aroF, aroG, aroH	DAHP synthetase I family	-2.20
3	gene9855	SPRG_08603	trpD	Glycosyl transferase family, a/b domain;Glycosyl transferase family, helical bundle domain	-2.29
4	gene7685	SPRG_20135	PAT, AAT	Aminotransferase class I and II;Prephenate dehydratase	-2.11
5	gene2736	SPRG_02249	TRP	Pyridoxal-phosphate dependent enzyme	-2.31
6	gene5831	SPRG_05285	E5.4.99.5	Prephenate dehydratase;ACT domain	-2.57
7	gene6777	SPRG_06062	trpE	chorismate binding enzyme;Anthranilate synthase component I, N terminal region	-2.13
8	gene831	SPRG_00707	E2.5.1.54, aroF, aroG, aroH	DAHP synthetase I family	-2.01
9	gene834	SPRG_00710	GOT2	Aminotransferase class I and II	-1.17
10	gene5624	SPRG_05079	TRP;	Tryptophan synthase alpha chain	-1.42
11	gene16260	SPRG_15106	hisC	Aminotransferase class I and II;Cys/Met metabolism PLP-dependent enzyme	-1.66
12	gene7858	SPRG_06771	TAT	Aminotransferase class I and II;Cys/Met metabolism PLP-dependent enzyme	3.24
13	gene9102	SPRG_08112	ARO1	EPSP synthase;3-dehydroquinate synthase;Type I 3-dehydroquinase;Shikimate kinase;Shikimate dehydrogenase substrate binding domain;Iron-containing alcohol dehydrogenase;Shikimate 5'-dehydrogenase C-terminal domain;Iron-containing alcohol dehydrogenase;Shikimate / quinate 5-dehydrogenase	-3.02
14	gene4328	hypothetical protein	E2.5.1.54, aroF, aroG, aroH	none	-1.90
